# A Sustainable and Alternative Packaging Approach for EU PDO Erzincan Tulum Cheese Affecting Food Safety, Proteolysis, Lipolysis, and Volatilome in Cheese: Sausage Casing

**DOI:** 10.1111/1750-3841.71111

**Published:** 2026-05-12

**Authors:** Abdurrahman Çelik, Ali Tekin, Didem Şahingil, Ali Adnan Hayaloğlu

**Affiliations:** ^1^ Department of Food Engineering Inonu University Malatya Türkiye; ^2^ Food Processing Department, Keban Vocational School Fırat University Elazığ Türkiye

**Keywords:** cheese volatiles, free amino acids, proteolysis in cheese, raw sheep milk cheese, water vapor permeability

## Abstract

Erzincan Tulum cheese is highly appreciated by consumers and holds significant social, economic, and cultural importance in Türkiye. Its shelf life and quality are influenced by packaging type and ripening conditions. This study aimed to investigate the effects of three different packaging materials—vacuum packaging (VP); plastic barrels (PBs), used as the control packaging; and sausage casing (SC)—on the ripening quality and volatilome of Erzincan Tulum cheese over 150 days, considering physicochemical properties, proteolysis, lipolysis, volatile compounds, and microbiological aspects. Coliform bacteria were not detected after Day 90 in VP and SC cheeses and after Day 120 in PB. Controlling water vapor permeability proved critical for maintaining moisture balance and extending shelf life. The moderate moisture barrier provided by SC helped preserve product quality and prevent microbial or chemical spoilage. Free amino acid accumulation was higher in SC (1327.30 mg/100 g) and VP (1533.46 mg/100 g) cheeses than in PB (999.02 mg/100 g) after 150 days, with all amino acids except histidine showing higher levels in SC and VP samples. Volatile compound distribution varied by packaging: carboxylic acids dominated in PB (36.97%) and SC (32.94%), esters (41.30%) in SC, and alcohols (14.66%) and hydrocarbons (15.35%) in VP cheeses. VP reached 63.92 mg TEAC/L and 29.17 mg Fe^2+^/L, whereas SC reached 64.20 mg TEAC/L and 26.04 mg Fe^2+^/L for 2,2‐azino‐bis‐(3‐ethylbenzothiazoline‐6‐sulfonic acid) (ABTS) and ferric reducing antioxidant power (FRAP) results as antioxidant activities. In conclusion, these results indicate that VP and SC packaging, as alternatives to PB for Erzincan Tulum cheese, preserved its unique characteristics during ripening and enhanced its volatile profile and antioxidant properties.

## 1 Introduction

Cheeses with protected designation of origin (PDO) status are traditional products made according to strict production standards that preserve their distinctive sensory characteristics, which are well recognized by consumers. To qualify for the PDO label, these cheeses must be made within a designated geographical region, utilizing locally sourced raw materials and adhering to specific regulations governing both their production and maturation processes (Lora et al. 2020).

Erzincan Tulum cheese, previously produced locally and on small‐scale farms, is now produced and exported on a larger scale. Tulum is the name given to cheeses typical of different regions such as Bingöl, Elazığ, and Akkaraman (Keser et al. 2024) in Türkiye, which are generally made in animal skins, with Tulum from goat‐skin bags being preferred. Erzincan Tulum cheese is manufactured during the period from May to September, when sheep and goat milk is most abundant (Tekin and Güler 2019; Tomar et al. 2020). The wide distribution of Erzincan Tulum cheese is due to its presence in all regions of Türkiye and many European countries, and it is a type of cheese with a PDO in the EU. Erzincan Tulum cheese is mostly sold in plastic barrels (PBs), whose protective and barrier functions are ensured using various plastic polymers (Zhang et al. 2022). Currently, the choice of packaging technology is primarily based on barrier properties, product type, and cost. Lamb or goat skins from animals less than 1‐year‐old are generally preferred as a packaging material due to their superior oxygen and moisture permeability (Tekinşen and Uçar 2007). In recent years, PBs have been widely used for packaging the cheese because of their ease of use and regulatory approval.

Apart from packaging Erzincan Tulum cheese in cloth, leather, and PBs, no studies have yet been conducted on packaging in vacuum packages or sausage casings (SCs). Using these types of packaging, especially permeable plastic casings, allows for the production of single‐portion products with low weight. The aim of this study is to contribute to the dissemination of knowledge about food safety and quality in packaging EU PDO Erzincan Tulum cheeses with alternative SC. In this context, packaging technology can influence the specific properties of portioned PDO cheeses, potentially resulting in varying shelf lives for products of identical cheese mass. Therefore, PDO Erzincan Tulum cheese, known for its distinctive qualities and significant market presence, was selected to examine the impact of different packaging materials on its ripening process. The physicochemical, proteolytic, microbiological, and volatilome of Erzincan Tulum cheese packaged with vacuum packaging (VP) and SC were analyzed and compared to those of cheese ripened traditionally in PBs.

## 2 Materials and Methods

### 2.1 Cheese Manufacture

Akkaraman sheep's milk was used to produce Erzincan Tulum cheese in the plateaus and highlands of Elazığ, Türkiye. The raw sheep's milk was filtered and coagulated using calf rennet (Mysecoren 200‐Maysa A.Ş., İstanbul, Türkiye) with a strength of 1:16,000 MCU/mL at 32–33°C for 90 min. After this time, the resulting curd was cut into 1 cm^3^ pieces, and the whey was removed. The curd was then placed in cotton cloths and left to rest at 22–24°C for 1 week. In this way, the remaining whey was almost completely removed. After this, the curd was cut into pieces of about 1 × 1 × 1 cm^3^ and placed into cotton bags to allow the whey to drain (Hayaloglu et al. 2007), and 2.5% coarse salt was added and mixed thoroughly. Once a homogeneous mixture was obtained, the curd was again placed in cotton cloths for filtering, and the first ripening process was carried out at 22°C for 3 days. The cheese removed for the second ripening process was shredded again and filled into an SC, a VP, and a PB, then subjected to a ripening process at 3–6°C and 75%–80% relative humidity for 150 days. Cheese production was carried out in two independent replicates.

### 2.2 Chemical Composition, Microbiology, and Water Vapor Permeability (WVP) of Cheese

The pH and titratable acidity of cheese samples were measured as described in Yılmaz et al. (2025).

Total solids content was assessed using a gravimetric approach, whereas fat content was analyzed by the Gerber method. Salt concentration was determined by Mohr titration, and total protein was quantified using the micro‐Kjeldahl method (Soltani et al. 2024). Enumeration of microorganisms: Yeasts and molds on potato dextrose agar (PDA) were incubated at 25°C for 5 days (Devi et al. 2016); total aerobic mesophilic bacteria were enumerated on plate count agar (PCA) following incubation at 33°C for 72 h; total coliforms were on violet red bile (VRB) agar incubated at 37°C for 48 h (Dimassi et al. 2020). Counts were calculated in at least three replicates and are expressed as log_10_ cfu (colony‐forming units)/g. The WVP of the SC packaging used for the cheese samples was determined using the gravimetric method according to ASTM E96/E96M (ASTM 2016). The thickness of the packaging was measured at 10 different locations using a digital micrometer (caliper) with a precision of 0.001 mm, and the average thickness was calculated (Larotonda et al. 2005). Five grams of desiccated CaCl_2_ were placed in a container with a diameter of 3 cm. The container was then sealed airtight over the opening of the packaging. The assembly was placed in a desiccator maintained at ambient temperature for 7 days, with a saturated NaCl solution to maintain 75% relative humidity. Weight measurements were taken every 24 h to monitor changes in weight. After 7 days, the WVP was calculated using the following equation:

$$\text{WVP}\left(g/\text{m s Pa}\right)=\frac{\Delta W\times I}{A\times \Delta t\times \Delta p}$$

where ΔW is the weight change in the package (g), I is the package thickness (m), Δt is the change in time (seconds), A is the exposed package area (m^2^), andΔp is the pressure difference (Pa).

### 2.3 Proteolysis

The levels of the pH 4.6‐soluble (pH 4.6‐SN) and 12% trichloroacetic acid–soluble nitrogen (TCA–SN) fractions were quantified using the micro‐Kjeldahl procedure. In addition, the peptide patterns of the pH 4.6‐soluble fractions and protein profiles of the pH 4.6‐soluble fractions were evaluated by urea‐polyacrylamide gel electrophoresis (urea‐PAGE), following the methodology by Sulejmani and Hayaloglu (2018). Total and individual free amino acid (FAA) concentrations in the pH 4.6‐SN fraction of cheese samples were analyzed using the reversed‐phase high‐performance liquid chromatography (RP‐HPLC) as described by Ayag et al. (2022).

### 2.4 Lipolysis (Acid Degree Value [ADV])

Lipolytic activity in cheese samples was assessed in accordance with the procedure described by Aguilar Uscanga et al. (2022). Briefly, 10 g of each cheese sample was accurately weighed into a lipolysis flask, and 10 mL of BDI solution prepared by dissolving 30 g of Triton X‐100 and 70 g of sodium tetraphosphate in 1 L of distilled water was added. The mixture was homogenized thoroughly to achieve complete dispersion and subsequently incubated in a hot shaking water bath at elevated temperature for 15–20 min. After a Gerber centrifugation, aqueous methanol solution (1:1, v/v) was introduced, and the samples were centrifuged again for 1 min. The fat collected in the neck of the flask was removed with a 1‐mL syringe and transferred to a small conical flask and weighed. Thereafter, 5 mL of oil solvent mixture (petroleum ether:*n*‐propanol, 4:1, v/v) and two to three drops of 1% phenolphthalein in ethanol were added, and the sample was titrated with 0.02 N alcoholic KOH until a clear pink color appeared. The volume of alcoholic KOH spent ($V_{\mathrm{sample}}$) was used for the calculation of ADV as given in the following formula:

$$\mathrm{ADV}:\frac{V_{\mathrm{sample}}\;\left(\mathrm{mL}\right)-V_{\mathrm{blank}}\;\left(\mathrm{mL}\right)\;\times \;0.02\times 100\;}{\mathrm{sample}\;\mathrm{amount}\;\left(g\right)}$$

The level of lipolysis was determined as meq KOH/100 g oil.

### 2.5 Antioxidant Activity

The antioxidant capacity of the cheese samples was evaluated using the ABTS (2,2‐azino‐bis‐(3‐ethylbenzothiazoline‐6‐sulfonic acid)) and FRAP (ferric reducing antioxidant power) assays, following the methodology described by Incili et al. (2023). Analyses were performed on the pH 4.6‐SN fraction, and absorbance measurements were obtained using a UV–visible spectrophotometer (Shimadzu, UV‐1800, Kyoto, Japan) at 734 nm for the ABTS assay and 593 nm for the FRAP assay. Antioxidant activity values derived from the ABTS method were expressed as Trolox equivalent antioxidant capacity (mg TEAC/L), whereas FRAP results were reported as mg Fe^2+^ equivalent/L.

### 2.6 Volatiles

The volatile compound composition of Erzincan Tulum cheese was analyzed by headspace solid‐phase microextraction (SPME) coupled with gas chromatography–mass spectrometry (GC–MS) using a Shimadzu QP2010 system (Japan), in accordance with the procedure reported by Sulejmani et al. (2020). Cheese samples (3.0 g) were placed in 15 mL glass vials and analyzed monthly in at least three replicates. Vials containing samples equilibrated at 40°C for 30 min. Volatile extraction was carried out by exposing a 2 cm SPME fiber coated with Divinlybenzene (DVB)/carboxen/Polydimethylsiloxane (PDMS) (50/30 µm; Supelco) to the vial headspace at 40°C for 30 min, followed by manual desorption in the GC–MS injector. Helium served as the carrier gas at a constant flow of 1.0 mL/min. Separation of volatile compounds was achieved using a DB‐Wax capillary column (60 m × 0.25 mm × 0.25 µm; J&W Scientific). Identification of compounds was based on mass spectral comparisons with the Wiley 9 (2005) and NIST14 (2014) databases. The results were given by dividing the peak areas of the compounds obtained from the device by 10^6^.

### 2.7 Statistical Analyses

All statistical analyses were conducted using SPSS software (version 16.0 for Windows). Differences among chemical and microbiological parameters were assessed by one‐way analyses of variance (ANOVA), and mean values were separated using Duncan's multiple range test at a significance level of *p *< 0.05. Homogeneity of variance among groups was verified using Levene's test (test for homogeneity of variance in SPSS), confirming that the assumption of equal variances was satisfied. Volatile compounds and FAAs were assessed using heat maps and PCA in the R project for statistical computing version 4.0 (https://www.r‐project.org). program. All analyses were performed in two independent trials, each conducted in triplicate.

## 3 Results and Discussions

### 3.1 Compositional and Microbiological Analyses

The highest pH values for PB samples were recorded as 4.71 on the first day and 5.49 on the 120th day of ripening, respectively (Table 1). The increase in pH observed during ripening is closely associated with microbial activities, particularly the conversion of lactic acid to ethanol by yeasts and the release of ammonia by proteolytic microorganisms (Galli et al. 2019; Łopusiewicz et al. 2020). This rise in pH supports the growth of the total number of mesophilic aerobic bacteria (TMAB) by creating a less restrictive environment for many mesophilic aerobic bacteria. Conversely, the decline in coliforms and the lower yeast and mold counts—especially in cheeses ripened in SC packaging—suggest that the reduced oxygen permeability and more controlled moisture balance provided by this material partially suppress the microbial proliferation that would otherwise be promoted by the increasing pH. However, this increase may also result from enhanced buffering capacity due to protein breakdown and the release of acids from the packaging (Talevski et al. 2017). The results obtained were similar to those for Mascarpone‐type cheese samples, where an increase in pH was also observed (de Almeida et al. 2018). The difference in titratable acidity of Erzincan Tulum cheese was significant (*p *< 0.05) on Day 150, whereas no difference was observed on the other storage days, with values ranging from 0.81% to 1.31% (Table 1). A significant difference was found in the moisture levels of Tulum cheese ripened in three different packaging systems (on Days 1, 60, and 90; *p *< 0.05). Regarding packaging material, the average moisture content of cheese ripened in SCs remained stable throughout storage and was higher than that of cheese ripened in plastic and VP (Table 1). This may be because cheese packaged in SCs helps Erzincan Tulum cheese retain its moisture during ripening. The protein content of Erzincan Tulum cheese matured with different packaging materials ranged between 21.12% and 23.26%. The protein content of some Tulum cheeses matured in PBs was reported as 24% for Muş Tulum (Rençber and Çelik 2021) and 21.37% for Kargı Tulum (Dinkçi et al. 2012). Salt in the cheese matrix affects the microbial dynamics and enzyme activity of cheese and also reduces the moisture content. No significant differences were found between the salt levels of Erzincan Tulum cheese, except on the 150th day (% salt 1.11–1.93).

**TABLE 1:** Chemical composition and pH of EU protected designation of origin (PDO) Erzincan Tulum cheeses ripened in various packages during 150 days of ripening.

		Cheeses		
Parameters	Days	PB	VP	SC
pH	1	4.71 ± 0.02^b,A^	4.44 ± 0.03^a,A^	4.63 ± 0.02^b,A^
	30	5.12 ± 0.05^a,C^	4.73 ± 0.00^b,C^	4.67 ± 0.01^b,AB^
	60	4.85 ± 0.01^c,B^	4.67 ± 0.01^a,B^	4.74 ± 0.01^b,B^
	90	4.79 ± 0.03^ab,B^	4.82 ± 0.01^b,D^	4.7 ± 0.05^a,C^
	120	5.49 ± 0.03^c,D^	4.69 ± 0.01^a,B^	4.77 ± 0.02^b,BC^
	150	4.86 ± 0.01^a,B^	4.87 ± 0.01^a,D^	4.86 ± 0.01^a,C^
Titratable acidity (% of lactic acid)	1	0.81 ± 0.00^A^	0.95 ± 0.19^A^	0.86 ± 0.06^A^
	30	1.26 ± 0.00^D^	1.31 ± 0.06^C^	1.26 ± 0.00^BC^
	60	1.17 ± 0.00^C^	1.22 ± 0.06^BC^	1.17 ± 0.00^B^
	90	1.22 ± 0.06^C^	1.26 ± 0.00^BC^	1.22 ± 0.06^BC^
	120	1.26 ± 0.00^D^	1.22 ± 0.06^BC^	1.31 ± 0.06^C^
	150	1.09 ± 0.01^ab,B^	1.04 ± 0.03^a,AB^	1.15 ± 0.03^b,B^
Moisture (%)	1	42.91 ± 0.12^b,C^	41.53 ± 0.37^a,A^	42.98 ± 0.31^b^
	30	42.89 ± 0.1^C^	41.67 ± 0.8^A^	42.94 ± 0.2
	60	42.93 ± 0.12^ab,C^	42.26 ± 0.28^a,AB^	43.44 ± 0.39^b^
	90	42.28 ± 0.2^a,B^	43.14 ± 0.26^ab,B^	44.39 ± 0.98^b^
	120	41.19 ± 0.00^a,A^	42.93 ± 0.4^a,B^	43.08 ± 1.28^b^
	150	40.78 ± 0.51^a,A^	41.49 ± 0.48^a,A^	42.67 ± 0.32^b^
S/M (%)	1	2.59 ± 0.19^A^	2.82 ± 0.03^A^	2.79 ± 0.08^A^
	30	3.96 ± 0.18^C^	4.21 ± 0.08^AB^	3.68 ± 0.21^B^
	60	3.54 ± 0.01^B^	3.74 ± 0.17^B^	3.77 ± 0.03^B^
	90	3.6 ± 0.02^B^	3.8 ± 0.02^AB^	3.56 ± 0.26^B^
	120	3.69 ± 0.2^C^	3.95 ± 0.23^AB^	4.21 ± 0.07^C^
	150	4.31 ± 0.14^b,C^	4.23 ± 0.35^a,AB^	4.11 ± 0.03^a,BC^
Protein (%)	1	23.43 ± 0.7^b,B^	25.14 ± 0.08^c,B^	21.67 ± 0.14^a,AB^
	30	21.87 ± 1.03^A^	23.26 ± 1.2^AB^	22.62 ± 0.67^AB^
	60	22.36 ± 0.2^AB^	22.57 ± 1.7^A^	21.26 ± 1.13^B^
	90	22.23 ± 0.02^b,AB^	21.12 ± 0.08^a,A^	23.2 ± 0.52^c,B^
	120	22.45 ± 0.00^AB^	21.74 ± 0.61^A^	22.82 ± 0.00^AB^
	150	22.82 ± 0.27^AB^	21.29 ± 0.36^A^	21.14 ± 1.02^A^

*Note*: Different superscripts within the same row are differ (*p* < 0.05). Capital letters mean within a column with no differ (*p *< 0.05).

Abbreviation: PB, plastic barrel; S/M, salt‐in‐moisture; SC, sausage casing; VP, vacuum package.

**FIGURE 1:**
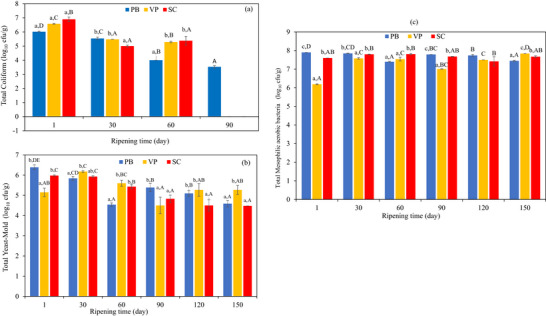
Microbiological amount (a) total coliform, (b) total yeast‐mold, (c) total mesophilic aerobic bacteria of EU PDO Erzincan Tulum cheeses used different packaging during 150 days of ripening (log_10_ cfu/g). Small letters (a–c) indicate differences among samples within the same storage day, whereas capital letters (A–E) indicate significant differences for the same sample on different storage days (*p *< 0.05). PB, plastic barrel; SC, sausage casing; VP, vacuum package.

Coliform bacteria, commonly used as hygiene indicators, ceased to grow after the 90th day of storage in the VP and sausage casing (SC) groups, and after the 120th day in the plastic barrel (PB) group, falling below detectable levels (<1.0 log_10_ cfu/g) (Figure 1a). Yeast and mould count generally decreased throughout the storage period, except in the VP cheese, where counts ranged from 4.50 ± 0.28 to 6.39 ± 0.12 log_10_ cfu/g. For yeast and mould levels, the SC group showed counts approximately 1 log lower than the other groups towards the end of storage (Figure 1b). This reduction is likely due to the lower oxygen availability within the sausage casing packaging. Packaging materials allow a certain degree of gas permeability, which depends on factors such as the type and thickness of the material, temperature, relative humidity, and the gas concentration gradient across the material surfaces (Iličić et al. 2016). The total number of mesophilic aerobic bacteria (TMAB) in Tulum cheeses increased, ranging from 6.19 ± 0.02 log_10_ cfu/g in the vacuum‐packed (VP) group to 7.9 ± 0.0 log_10_ cfu/g in the plastic barrel (PB) group (Figure 1c). These results are consistent with previous studies reporting an increase in mesophilic aerobic bacteria during the ripening period (Hayaloglu et al. 2007; Demir et al. 2023).

The WVP values of the SC packaging were found to range between 1.31 × 10^−11^ and 5.67 × 10^−11^ g/(m^2^ s Pa) (Table 2). Although the results showed an increasing trend, the packaging materials were found to provide a moderate moisture barrier. The retention of moisture during ripening and the absence of statistically significant differences among the Tulum cheese samples packaged in SC were consistent with the WVP results. This finding is crucial for maintaining the shelf life of Tulum cheese. Experimental data indicated that the increase in moisture content within the test chamber was associated with mass gain fluctuations observed over a 7‐day period due to the hygroscopic properties of CaCl_2_. As the diffusion rate increased, a nonlinear rise in mass gain was observed. According to ASTM E96 (ASTM International 2016), such a nonlinear increase is typical until equilibrium is reached. Hu et al. (2024) reported that reducing WVP contributes to prolonging the shelf life of foods coated with edible films and helps prevent microbial and chemical deterioration. This finding supports that the moderate moisture barrier observed in this study may play a significant role in maintaining the quality and stability of Tulum cheese during storage.

**TABLE 2:** Water vapor permeability of sausage casing package used as a packaging material for EU protected designation of origin (PDO) Erzincan Tulum cheese.

Weight no	Container weight (g)	Δ*W* (g)	Water vapor permeability (g/m^2^ s Pa) × 10^−11^
T0	260.24	0.00	0.00
T1	260.29	0.05	1.31
T2	260.30	0.06	1.56
T3	260.32	0.08	2.13
T4	260.35	0.12	2.95
T5	260.38	0.14	3.56
T6	260.46	0.22	5.67

*Note*: Δ*W*: weight change in the package (g).

### 3.2 Proteolysis

#### 3.2.1 Soluble Nitrogen Fractions

**FIGURE 2:**
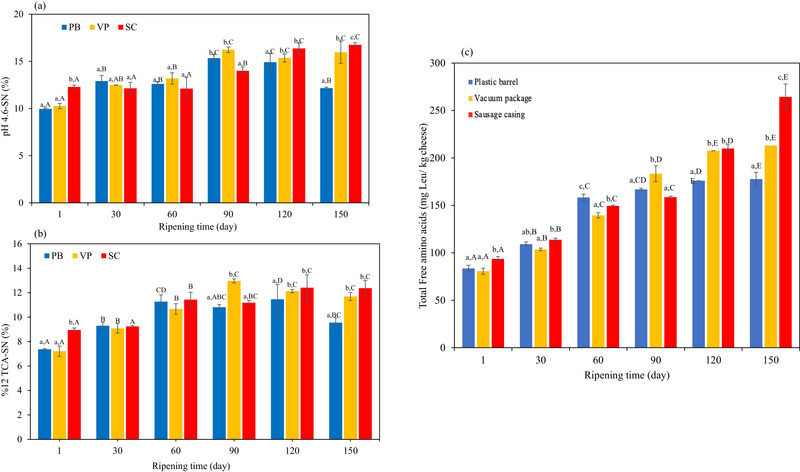
(a) pH 4.6‐SN and (b) 12% TCA–SN of EU PDO Erzincan Tulum cheeses. (c) Total free amino acid value (mg Leu/kg cheese) of EU PDO Erzincan Tulum cheeses. Small letters (a–c) indicate differences among samples within the same storage day, whereas capital letters (A–E) indicate significant differences for the same sample on different storage days (*p *< 0.05). PB, plastic barrel; SC, sausage casing; TCA–SN, trichloroacetic acid–soluble nitrogen; VP, vacuum package.

The levels of pH 4.6‐SN increased continuously from the first to the last day of storage. The ripening index is an important value because it indicates the proportion of pH 4.6‐SN in the total nitrogen in the cheese. The average levels of primary proteolysis, measured by the SN content at a pH of 4.6, gradually increased during storage regardless of the packaging conditions (Figure 2a). However, the increase in proteolysis was particularly pronounced in the samples packaged with SC. Across all groups and storage times, the lowest value (9.96% ± 0.09%) was observed on Day 1 in the PB group and the highest (16.76% ± 0.21%) on Day 150 in the SC group. Both ripening time and packaging significantly affected pH 4.6‐SN and TCA–SN values (*p *< 0.05), which generally increased during storage except for the PB sample on Day 150 (*p *< 0.05). On Day 120, the values were 11.46% ± 1.21% in the PB group, 12.97% ± 0.16% in the VP group, and 12.41% ± 1.05% in the SC group (Figure 2b). The lowest values were recorded at the beginning of storage in the samples packed in PBs (9.96% and 7.37%) and in the VP samples (10.26% and 7.21%), whereas the highest values were recorded at the end of storage in the VP samples (15.96% and 11.69%) and (16.76% and 12.37%). The TCA–SN value in the cheese samples packed with SC was higher than in those packed with other materials.

#### 3.2.2 Urea‐PAGE of pH 4.6‐insoluble and RP‐HPLC Peptide Profile of pH 4.6‐Soluble Fractions

**FIGURE 3:**
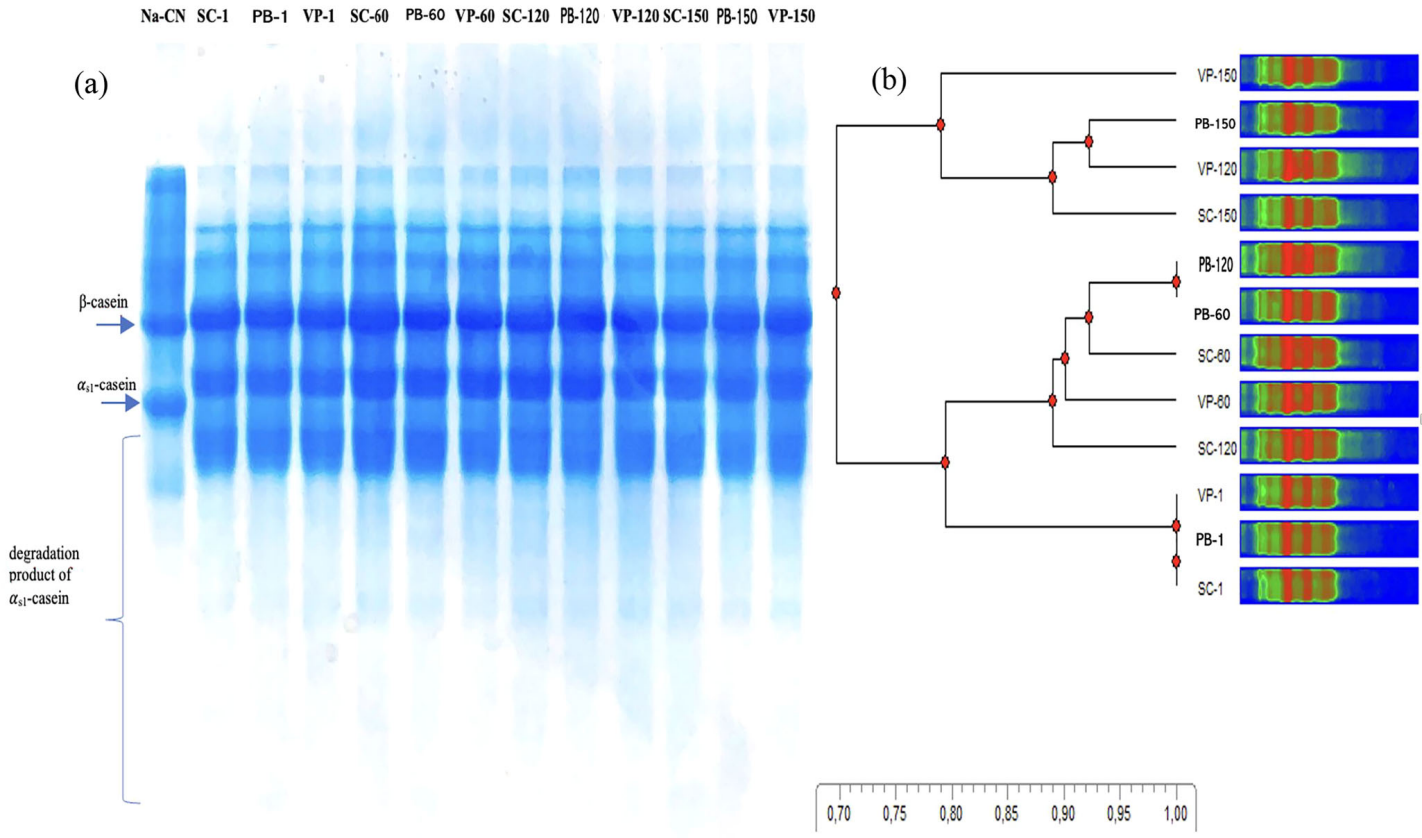
(a) Electrophoretic profile and (b) dendrogram of EU PDO Erzincan Tulum cheeses by urea‐PAGE electrophoresis. PB, plastic barrel; SC, sausage casing; VP, vacuum package.

**FIGURE 4:**
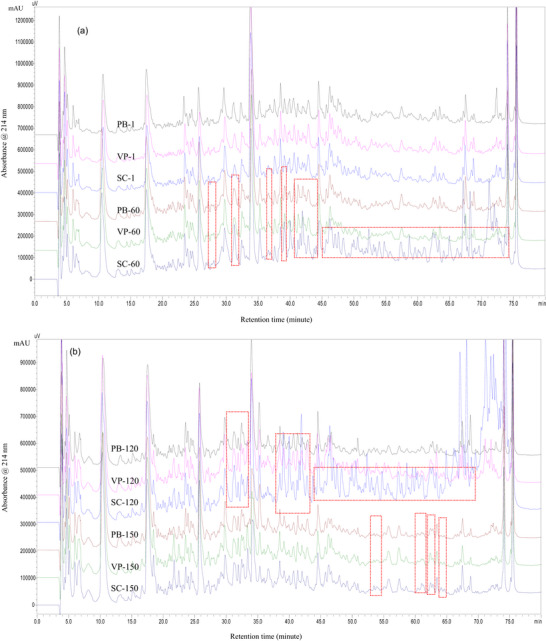
(a) Reversed‐phase high‐performance liquid chromatography (RP‐HPLC) of EU PDO Erzincan Tulum cheese samples (Days 1 and 60). (b) RP‐HPLC of EU PDO Erzincan Tulum cheese samples (Days 120 and 150). PB, plastic barrel; SC, sausage casing; VP, vacuum package.

The urea‐PAGE patterns and RP‐HPLC peptide profiles of Erzincan Tulum cheese are shown in Figures 3a,b and 4a,b. Changes in casein fractions and peptides were observed throughout ripening. No significant differences were observed in protein bands by urea‐PAGE; however, α_s1_‐ and β‐casein hydrolysis was greater in cheese ripened in SC. The dendrogram (Figure 3b) shows that VP samples at Day 60 formed a distinct cluster from others. Sample gels of SC after 60 days, PB after 120 days, VP after 120 days, and SC after 150 days were grouped together. Additionally, the VP and PB samples at 150 days of ripening were distinct from the others and grouped together in the same cluster. These electrophoretic results are consistent with other proteolysis parameters, such as pH‐4.6‐SN, TCA–SN, HPLC peptide profiles, and FAA content.

Proteolysis was also investigated by analyzing the peptide profile of cheese samples using RP‐HPLC. The amino acid composition mainly affects the retention time of small peptides (<15 residues), whereas larger peptides and proteins are influenced by factors like conformation and molecular weight (Uğur and Öner 2023). Figure 4a,b shows peptide profiles from cheeses packaged with different materials on Days 1, 60, 120, and 150 of ripening. On Day 1, the RP‐HPLC chromatograms of all samples were similar, with most peptides eluting between 10 and 70 min. SC cheeses showed more peaks between 30 and 45 min, linked to α_s1_‐ and β‐casein degradation. By Day 60, distinct differences appeared among SC, PB, and VP cheeses, especially in the hydrophobic region. Larger peptides eluting between 45 and 75 min increased in SC cheeses on Day 120 but decreased by Day 150 (Figure 3b).

#### 3.2.3 Total and Individual FAAs

**FIGURE 5:**
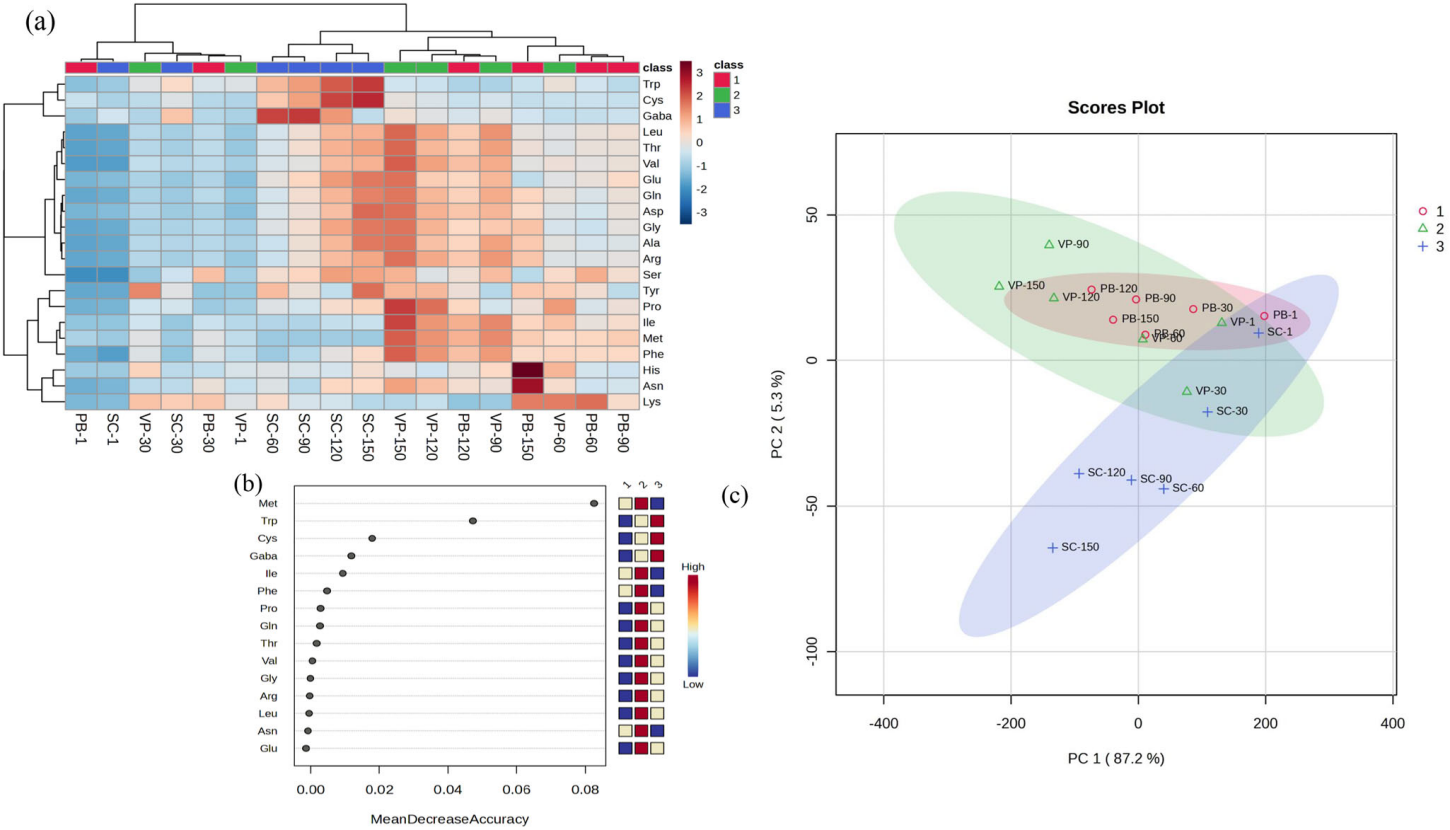
(a) Heatmap of individual free amino acid values in EU PDO Erzincan Tulum cheeses and (b) VIP profile of EU PDO Erzincan Tulum cheeses. (c) PLS‐DA plot of FAAs. PB, plastic barrel; SC, sausage casing; VP, vacuum package.

Proteolysis is a key change during cheese storage. On Day 1, FAA levels were similar in all samples, except in those packed in SCs, aligning with previous findings (Adámek et al. 2021, 2024). The most notable increase in total FAA occurred between Days 90 and 150, with levels approximately doubling in all samples (*p *> 0.05). During storage of cheese samples in different packaging materials, differences in TFAA concentrations were observed. Both ripening time and sample type had statistically significant effects (*p *< 0.05). The FAA concentration gradually increased in all cheese samples during the storage period. As shown in Figure 2c, proteolysis levels varied for cheeses stored in three different types of packaging, with values significantly higher in the SC on Day 150 of storage (*p *< 0.01). In addition, the proteolysis values at the end of the storage period were higher for VP and SC‐wrapped samples than for those packaged in PBs. These results indicate a significant increase in proteolysis between Days 0 and 150. In addition, the different packaging methods and storage time had a significant effect (*p *< 0.05) on proteolysis levels. The influence of different packaging materials on the proteolysis has been previously studied. For example, Hayaloglu et al. (2007) observed slightly lower TFAA levels (not significant) in cheeses stored in PBs compared to those in goat skin bags, although the differences in material permeability can affect water activity during ripening. Similar conclusions were drawn by Adámek et al. (2024), who found that higher moisture content and water activity were associated with increased proteolysis. These findings are further supported by studies from Gürsoy (2009), Simona (2012), and Thodis et al. (2023). Proteolysis, particularly the release of FAAs and low‐molecular‐weight peptides, is primarily driven by the activity of chymosin as well as starter and non‐starter bacterial enzymes (El Soda et al. 2000). Therefore, factors such as high humidity and low pH—both of which can influence chymosin activity—may contribute to changes in FAA levels (Atallah et al. 2022). In this study, the extent of proteolysis in Tulum cheese was monitored by measuring individual FAA concentrations on Days 1, 30, 60, 90, 120, and 150 of ripening (Figure 5). The total concentration of individual FAAs increased in all cheeses in the group. Cheeses ripened in VP and SCs had higher concentrations of most FAAs, except for Lys. On the 150th day, the Lys concentration in VP decreased from 15.60 mg/100 g to 13.20 mg/100 g, whereas the corresponding values were 17.39 and 13.44 mg/100 g in SC and 7.96 and 40.05 mg/100 g cheese in PB, respectively. A decrease in FAAs was observed in PB cheese, except for eight FAAs (Asn, Gly, Her, Arg, Thr, Ala, Tyr, and Trp). The profile of FAAs (Figures S1 and S2 and Table S1) shows that Glu, Arg, Val, Tyr, Ile, Leu, and Phe are the major FAAs, and the total concentration of individual FAAs was higher in cheeses ripened in VP (1382.46 mg/100 g cheese) than in those ripened in PB (847.03 mg/100 g cheese) and SC (1159.20 mg/100 g cheese). Hayaloglu et al. (2007) also reported that the most abundant FAAs in Tulum cheeses were Glu and Leu. These results are consistent with those of Ayag et al. (2022), who reported that the major FAAs in Tulum cheeses were Arg, Tyr, Ile, Leu, and Trp.

Although the effect of VP on proteolysis and lipolysis during cheese ripening is still under debate, studies have shown that it can affect biochemical processes (Hayaloglu et al. 2012; Miloradovic et al. 2018). This effect is likely due to the anaerobic conditions created by VP, which can alter the microbial population and, consequently, the activity of proteolytic enzymes (Domingues Galli et al. 2024; Nogueira et al. 2021). To evaluate similarities, differences, and trends among samples, multivariate techniques, such as partial least squares discriminant analyses (PLS‐DA) and heat map analyses, are commonly used. These tools allow for a comparative and visual interpretation of biochemical changes between samples. These analyses were applied to the complete data set (18 samples). In the amino acid profile shown in Figure 5a, the samples were grouped into three main clusters, specifically according to storage days. PLS‐DA was conducted to determine the relationships between the FAA contents and the samples (Figure 5c). PLS‐DA revealed that the Erzincan Tulum cheese samples could be categorized into three clusters on the basis of PC1 and PC2, which explained 87.2% and 5.3% of the total variance, respectively. PC1 (horizontal plot) had the greatest weight and resulted in the most significant differentiation of the samples along this plot.

As shown in Figure 5c, a gradual change in profile from left to right in the PLS‐DA plot was observed during the ripening period, indicating continuous fermentation. The most pronounced changes were observed in the VP and SC samples. Positive loadings in PC1 include the abundance of amino acids in the samples. Fresh cheeses (Day 1) had similar FAA content, located in the positive regions of PC1 and PC2. However, as storage time increased (especially Days 120 and 150), the FAA profiles changed, with samples shifting to the left and down along the PC1 and PC2 axes. Depending on the packaging type, SC‐type packaged samples (SC‐120, SC‐150) clustered in this area, indicating significant changes in FAA composition.

In the heat map, red and blue indicate higher and lower FAA levels, respectively. The super clustering divides the samples into three groups based on packing type: red (Class 1) PB samples, green (Class 2) VP samples, and blue (Class 3) SC samples; thus, samples of the same packing type are grouped together, showing similar amino acid profiles. As shown in Figure 5a, 20 amino acids were detected and categorized into two main groups and three subgroups on the basis of their amino acid content. The lower part of the dendrogram clearly shows that cheeses ripened under VP and in SC have similar FAA distributions. According to the upper dendrogram, the samples are divided into two main groups; the left main group consists of two subgroups, with PB‐1, SC‐1, VP‐30, and SC‐30 forming one subgroup, whereas PB‐30 and VP‐1 form the other. This group mainly includes samples from Days 1 to 30. The right main group is divided into three subgroups: SC‐60, SC‐90, SC‐120, and SC‐150 are in the first subgroup; VP‐150, VP‐120, PB‐120, and VP‐90 are in the second; and PB‐150, VP‐60, PB‐60, and PB‐90 are in the third. The VIP score graph generated by PLS‐DA analysis shows 15 amino acids with VIP scores greater than 1.0, which contribute most to separating the samples into clusters and are present in high proportions in these samples (Figure 5b). Among these 15 amino acids, Trp, Cys, and Gaba (in the SC sample); Met, Ile, and Phe (in the VP sample); and nine other amino acids (in the VP and SC samples, respectively) had the highest VIP scores. These results suggest that differences in FAA content between the cheeses are primarily due to the type of packaging and ripening time.

### 3.3 Lipolysis (ADV)

Various free fatty acids, which act as precursors for volatile compounds such as esters, aldehydes, and lactones through lipolysis in cheese, play a crucial role in flavor development (Lopez et al. 2006). These fatty acids are primarily responsible for the characteristic slightly pungent and rancid flavor of Tulum cheese (Hayaloglu et al. 2007; Ozturkoglu‐Budak et al. 2018). Tulum cheese is produced from raw milk and therefore exhibits more intense lipolysis compared to cheeses produced from pasteurized milk. As it is produced without the use of starter cultures, the lipolysis in this type of cheese may be attributed to the milk's lipoprotein lipase and/or lipases synthesized by non‐starter lactic acid bacteria and secondary microorganisms (Tekin and Güler 2019). The ADV is commonly used to quantify the amount of free fatty acids in milk fat and serves as an indicator of the extent of lipolysis. During the ripening period, ADV increased significantly, ranging from 2.36 to 30.14 meq KOH/100 g oil. Tekin and Güler (2019) also reported that as a result of their analyses of Tulum cheese produced from raw milk, the amounts of palmitic and oleic acids, which were found in the highest concentrations, showed an increase of approximately 10 folds by the end of storage. The change in ADV values determined in the present study can be considered similar to that observed in the aforementioned study.

**FIGURE 6:**
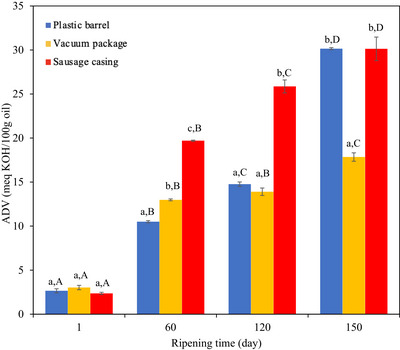
ADV value (meq KOH/100 g oil) of EU PDO Erzincan Tulum cheeses. Small letters (a–c) indicate differences among samples within the same storage day, whereas capital letters (A–D) indicate significant differences for the same sample on different storage days (*p *< 0.05).

The highest ADV was recorded in Tulum cheese ripened in PB and SC at the end of ripening (30.14 and 30.13 meq KOH/100 g oil), which were statistically higher than the values observed in VP packaging types. Both ripening time and packaging material exerted significant effects on lipolysis values (*p *< 0.05). Previous studies on Tulum cheese have also reported that lipolysis increases during storage (Ozturkoglu‐Budak et al. 2018; Karagol and Tarakci 2024). On the other hand, especially on Day 150, lipolysis values of vacuum‐packaged cheeses were lower compared to those packaged in SCs and PBs (Figure 6). This situation was likely due to the presence of oxygen within the packaging. The absence of oxygen in vacuum‐packaged samples and the oxygen permeability of SCs and PBs appear to have affected lipolytic activity. These findings are consistent with the observation that Tulum cheeses matured in the highly oxygen permeabile animal skin packaging have a higher fatty acid content than those matured in PBs (Tekin and Güler 2019).

### 3.4 Antioxidant Activity

The ABTS and FRAP methods were used to determine the antioxidant activity of cheese samples. Table 3 presents the results of both analyses for cheeses ripened in three different packaging types. ABTS is a decolorization test that can be applied to both lipophilic and hydrophilic antioxidants (Re et al. 1999). On the basis of the ABTS analyses results, VP and SC samples exhibited the highest antioxidant activity, with values of 63.92 ± 0.85 and 64.2 ± 0.31 mg TEAC/L, respectively, on the 150th day. PB Tulum cheese followed with a potential antioxidant activity of 58.12 ± 0.48 mg TEAC/L (Table 3). ABTS radical scavenging activity increased significantly (*p *< 0.05) on the 150th day of ripening in VP and SC samples; however, the increase became insignificant (*p *< 0.05) after the 30th day in the PB sample. Proteolysis leads to the release of small peptides, which become more prominent as ripening progresses (Figure S2). The synthesis of water‐soluble peptides in ripened cheese has been closely linked to antioxidant properties (Torres‐Salas et al. 2024). In particular, the N‐terminal region of food‐derived antioxidant peptides often contains hydrophobic amino acids such as Val and Leu, as well as residues such as Pro, Cys, His, Tyr, Trp, Phe, Gly, and Met (Yang 2024). On the basis of the data in Table S1, the antioxidant activity in Tulum cheese extracts may be influenced by the presence of these amino acids. Different cheese packaging methods were also found to affect potential antioxidant activity. Factors such as ripening period, conditions, and fermentation processes significantly impact the antioxidant activity of cheeses (Santiago‐López et al. 2018). Consistent with previous findings (Öner and Sarıdağ 2018), antioxidant activity of Tulum cheese increased toward the end of ripening. However, in PB cheese, antioxidant activity did not change significantly after Day 60 (*p *> 0.05). Similarly, Bottesini et al. (2013) found that antioxidant activity remained almost constant throughout the ripening period. They stated that the amino acid side chains responsible for antioxidant activity were able to maintain their capacity during ripening. According to both methods, the antioxidant capacity values of VP and SC samples were higher than those of PB samples. VP cheese had the strongest potential ferric ion reducing activity, with 29.17 ± 0.74 mg Fe^2+^ equivalent/L (Table 3). FRAP values for SC and PB were 26.04 ± 0.10 and 12.76 ± 0.37 mg Fe^2+^ equivalent/L, respectively. PB cheese showed significantly lower potential antioxidant activity than the other varieties in the FRAP experiment. VP is also recognized for reducing oxidation reactions by limiting exposure to oxygen (Ramírez‐Rivas et al. 2022).

**TABLE 3:** Antioxidant activity of EU protected designation of origin (PDO) Erzincan Tulum cheeses ripened in different packages during 150 days of ripening.

		Cheeses		
Parameter	Days	PB	VP	SC
ABTS assay (mg TEAC/L)	1	52.37 ± 1.05^a,B^	58.59 ± 0.79^b,A^	56.17 ± 1.64^ab,A^
	60	56.03 ± 0.07^a,C^	58.78 ± 0.41^ab,A^	60.46 ± 1.93^b,B^
	120	56.26 ± 0.21^a,C^	58.77 ± 1.74^b,A^	60.54 ± 0.93^b,B^
	150	58.12 ± 0.48^a,C^	63.92 ± 0.85^b,B^	64.2 ± 0.31^b,C^
FRAP assay (mg Fe^2+^/L)	1	11.98 ± 0.02^a,A^	18.49 ± 0.16^b,A^	26.56 ± 0.01^c,B^
	60	14.58 ± 0.01^a,AB^	23.96 ± 0.21^b,B^	23.44 ± 0.99^b,A^
	120	11.98 ± 0.0^a,A^	24.48 ± 0.73^b,B^	24.23 ± 0.0^b,A^
	150	12.76 ± 0.37^a,B^	29.17 ± 0.74^c,C^	26.04 ± 0.10^b,B^

*Note*: Different superscripts within the same row are differ (*p *< 0.05). Capital letters mean within a column with no differ (*p *< 0.05).

Abbreviations: ABTS, 2,2‐azino‐bis‐(3‐ethylbenzothiazoline‐6‐sulfonic acid); FRAP, ferric reducing antioxidant power; PB, plastic barrel; PDO, protected designation of origin; SC, sausage casing; VP, vacuum package.

### 3.5 Volatiles by SPME/GC–MS

**FIGURE 7:**
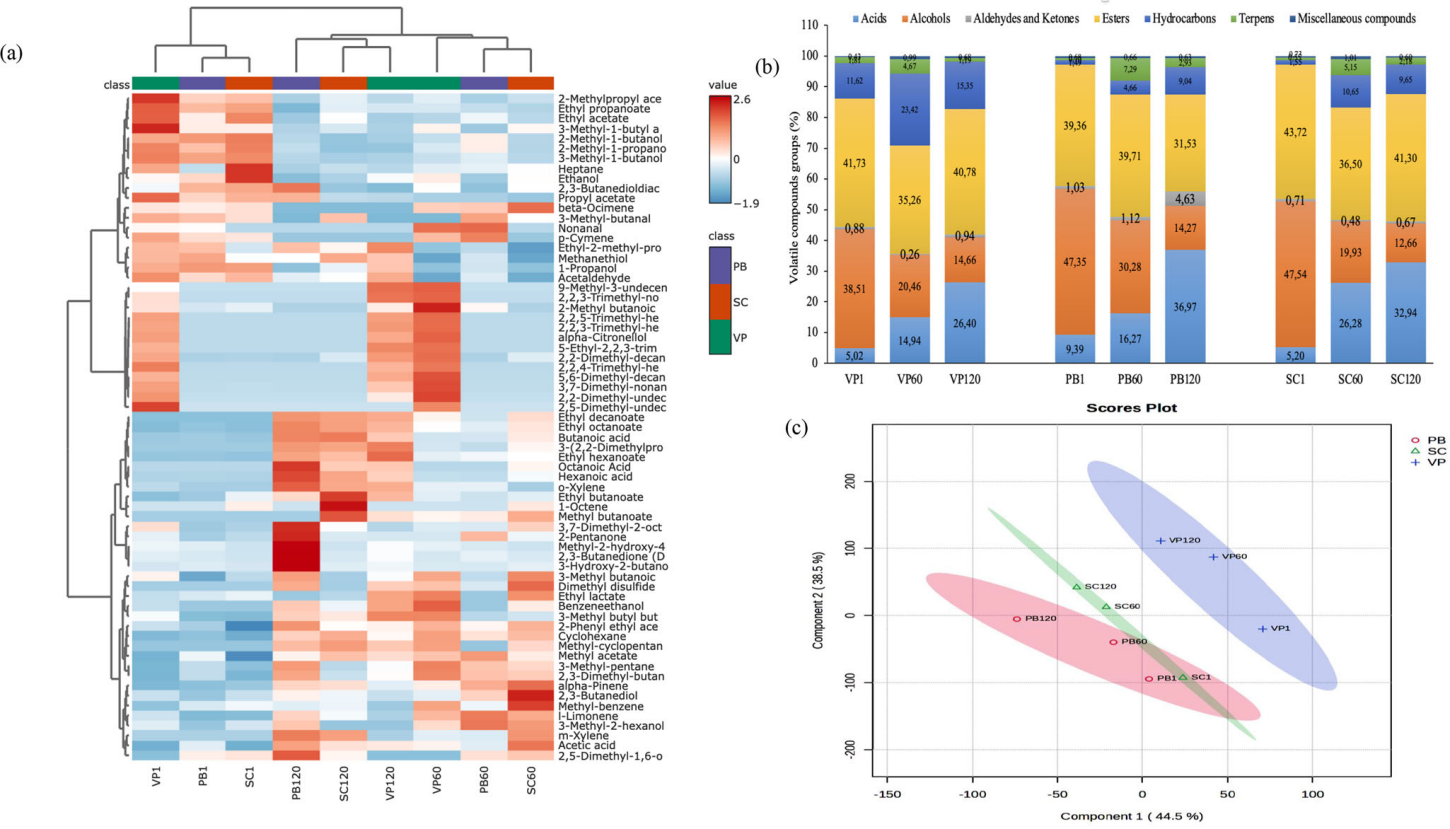
(a) Volatile compounds heatmap of EU PDO Erzincan Tulum cheeses and (b) volatile compounds groups (%) in EU PDO Erzincan Tulum cheeses. (c) Volatile aroma profile PCA of EU PDO Erzincan Tulum cheeses. PB, plastic barrel; SC, sausage casing; VP, vacuum package.

A total of 67 volatile compounds were identified in the cheese samples using the SPME technique, including acids (6), alcohols (8), aldehydes and ketones (6), esters (17), hydrocarbons (21), terpenes (5), and various other compounds (4) (Tables S2–S8). Although hydrocarbons (21) represented the largest group by number, the characteristic volatile compounds of Tulum cheese were alcohols and esters, which accounted for the highest percentage of total volatile compounds (Figure 7b). Alcohols have a significant influence on the flavor of Tulum cheese, and the use of raw milk in its production can result in a high alcohol concentration (Hayaloglu et al. 2007). Fernández‐García et al. (2004) reported a significant difference in the alcohol content of Manchego cheese produced from raw versus pasteurized sheep's milk, with raw milk cheese exhibiting higher alcohol content. The presence of non‐starter lactic acid bacteria, enterococci, and yeasts in raw milk has been identified as a contributing factor to increased alcohol production (Tekin and Guler 2021). In the present study, the total alcohol content was found to decrease during storage. Similarly, in Erzincan Tulum (Cakir et al. 2016) and Manchego (Fernández‐García et al. 2004) cheeses made from raw milk, the alcohol content, which was high at the beginning of storage, decreased during storage. Soltani et al. (2024) found that in cheese made from raw sheep's and goat's milk, sheep's cheese has a higher alcohol content, and the alcohol content decreases with aging. 3‐Methyl‐1‐butanol, a product of leucine amino acid catabolism (Tekin and Guler 2021), and ethanol, which is produced by lactose fermentation, amino acid catabolism, or from acetaldehyde (Tekin and Hayaloglu 2023), were the most frequently identified compounds in the cheese samples (Table S2).

Esters, which impart a fruity flavor to cheese and are formed by alcoholysis and esterification (Hayaloglu et al. 2007), were another group of volatile compounds detected at high levels in all cheese samples throughout storage. Ethyl acetate, ethyl butanoate, ethyl hexanoate, and 3‐methyl‐1‐butyl acetate had the highest peak areas among the esters (Table S3). Esters are produced by the esterification of the corresponding alcohols and acids, and the levels of these esters parallel those of the corresponding acids, which increased significantly in the cheese samples, especially at the end of storage. The acids contribute to the development of a pungent and unique aroma in Tulum cheeses as ripening progresses. The proportion of acids in the total volatile compound composition increased significantly with ripening (Figure 7b). Tekin and Guler (2021) reported that short‐chain linear acids such as butanoic, hexanoic, octanoic, and decanoic acids in Karaman Tulum cheese increased steadily during storage. C4–C8 acid compounds also increased in Lighvan cheese made from raw sheep and goat milk, particularly after 90 days of ripening (Soltani et al. 2024). These findings are consistent with the increases in butanoic, hexanoic, and octanoic acids observed after 60 days, and especially after 120 days, in the present study. Short‐ and medium‐chain free fatty acids not only contribute directly to cheese flavor but also serve as precursors for the formation of flavor compounds such as secondary alcohols, esters, methyl ketones, alkanes, and lactones (Tekin and Güler 2019). Table S4 shows that the total amount of acidic compounds, which was similar at the beginning, increased more in the SC samples after 60 days, whereas the PB sample reached the highest acid content after 120 days, followed by the SC and VP samples. This result is consistent with the ADV results indicating the extent of lipolysis (Figure 6).

Accordingly, the results show that vacuum‐packaged (VP) samples exhibited lower lipolytic activity compared to samples in other packaging types, and therefore, the formation of acidic compounds was less, especially at the end of storage, compared to other samples (Table S4). Another group of volatile compounds found abundantly in cheese samples was hydrocarbons (Table S6). Hydrocarbons are produced by the breakdown of fatty acids and chlorophyll from plants consumed by animals, and due to their high perception threshold, most hydrocarbons do not contribute to cheese flavor (Tekin and Guler 2021). However, they can form as secondary products of lipid autoxidation and are precursors of aromatic compounds (Hayaloglu et al. 2007). On the basis of these explanations, the low number of hydrocarbons at the beginning of storage in the PB and SC samples could originate from the milk used as raw material, depending on animal nutrition, and the increase with ripening in both cheeses could be related to lipolytic activity. In the VP sample, high levels of hydrocarbon compounds were detected from the beginning of storage and increased further with ripening. In relation to the ADV results, the presence of the highest amount and number of hydrocarbons in the VP sample, which had the lowest degree of lipolysis, could indicate the presence of a sufficient amount of free fatty acids, albeit to a lesser extent than in the other samples, and a high degree of degradation of these fatty acids in the vacuum environment. Flavor development in cheese occurs through biochemical reactions such as glycolysis, proteolysis, and lipolysis, which increase with ripening in cheeses made from raw milk such as Tulum cheese (Tekin and Güler 2019).

The presence of terpenes in dairy products is directly related to the animals’ diet. Terpenes, which are abundant in the milk of animals fed in mountainous regions and pastures, play an important role in cheese flavor (Tekin and Guler 2021). In this study, five different terpene compounds were identified, with l‐limonene being the most abundant (Table S7). The concentration of l‐limonene increased in all samples on the 60th day of ripening, then it decreased again on the 120th day. l‐Limonene is not a natural fermentation product in cheese, and its presence is related to the diet of the animals from which the milk is obtained. After l‐limonene, the terpenes detected in the highest amounts were 𝛽‐ocimene and 𝛼‐pinene. Similarly, these compounds also increased in all samples on the 60th day of storage; pinene decreased on the 120th day, and 𝛽‐ocimene was completely depleted. Tekin et al. (2025) found that terpenes decreased at the end of storage and suggested that this might be related to the lactic acid bacteria and yeasts present in the samples. This is because lactic acid bacteria, yeasts, and fungi have been reported to have the ability to modify and biosynthesize terpenes (Degenhardt et al. 2009). Additionally, it has been reported that the reduced terpenes can be metabolized to other hydrocarbon derivatives (Tekin and Guler 2021).

Heatmap and PLS‐DA graphs were created to observe how the volatile compounds in the samples were affected by ripening time and treatment (Figure 7a,c). Examination of Figure 7a shows that the samples are categorized into two groups on the basis of storage time: fresh and matured cheese. The ripened cheeses were further divided into two groups, with the 60‐ and 120‐day ripened cheeses forming separate clusters. This indicates that the biochemical changes, as previously mentioned, have a significant impact on the composition of the volatile compounds in the samples. Within these groups, the VP sample is separated from the PB and SC samples at the beginning and middle of storage, and the PB sample is separated from the other two cheeses at the end of 120 days. This may be due to the higher levels of acid compounds and the increased aldehyde and ketone levels found in the cheese matured in PBs on the last day of storage (Figure 7b). In the PLS‐DA diagram, Component 1 (45.3%) and Component 2 (37.7%) account for 83% of the total variance. Although the samples are essentially divided into three groups, the PB and SC cheeses are close together in the graph at the beginning of storage (Figure 7c), as in the heatmap. The VP sample is located on the right side of the diagram in relation to Component 1, whereas the PB and SC samples are positioned on the left. Regarding Component 2, the matured VP and SC cheeses are located in the upper part of the graph, whereas the matured PB cheese is positioned at the bottom. According to these results, ripening time has a significant effect on the volatile compounds of the cheese samples, and different packaging treatments also influence the composition of the volatiles in the cheese.

## 4 Conclusion

This study demonstrated that packaging type significantly influenced the ripening quality, biochemical development, and microbial stability of Erzincan Tulum cheese over 150 days, with SC and VP outperforming traditional PBs. The moisture control and moderate water WVP provided by SC limited microbial and chemical spoilage and eliminated coliforms at earlier stages of ripening. This resulted in higher FAA accumulation in SC and VP cheeses compared to PB, supporting more advanced proteolysis and lipolysis. Differences in volatile aroma profiles due to packaging—specifically, higher ester levels in SC and increased alcohol and hydrocarbon content in VP—contributed to the development of a more desirable flavor. In practice, the use of SC and VP offers the potential for single‐serving production formats and a reduced reliance on bulky PB materials, supporting cost efficiency and broader market compatibility while preserving the unique PDO characteristics of Erzincan Tulum cheese. In conclusion, SC and VP represent promising, modern alternatives that enhance the technological and functional properties of EU PDO Erzincan Tulum cheese while maintaining its traditional identity. Future research should focus on optimizing various SC materials, such as collagen, cellulose, or natural polymer‐based edible and protective casings, by evaluating their industrial application costs and comparing their permeability properties to maintain or improve for EU PDO Erzincan Tulum cheese quality during ripening. The focus should also be on validating these findings through industrial‐scale trials and implementing expanded consumer acceptance studies to support commercialization.

## Author Contributions

**Abdurrahman Çelik**: investigation, writing – original draft. **Ali Tekin**: investigation, writing – review and editing. **Didem Şahingil**: writing – review and editing, project administration. **Ali Adnan Hayaloğlu**: writing – review and editing, methodology.

## Funding

This study was financially supported by the Scientific Research Project Unit of Inonu University, Malatya, Türkiye, under project number FYL‐2022‐3134.

## Conflicts of Interest

The authors declare no conflicts of interest.

## Supporting information




**Supplementary Figures**: jfds71111‐sup‐0001‐Suppl. Figures R1.pptx

Table S1 Levels of individual free amino acids (mg/100 g cheese) in EU PDO Erzincan Tulum cheeses ripened in different package materials.
Table S2 Alcohols in EU PDO Erzincan Tulum cheeses ripened in different package materials.
Table S3 Esters in EU PDO Erzincan Tulum cheeses ripened in different package materials.
Table S4 Acids in EU PDO Erzincan Tulum cheeses ripened in different package materials.
Table S5 Aldehydes and ketones in EU PDO Erzincan Tulum cheeses ripened in different package materials.
Table S6 Hydrocarbons in EU PDO Erzincan Tulum cheeses ripened in different package materials.
Table S7 Terpenes in EU PDO Erzincan Tulum cheeses ripened in different package materials.
Table S8 Miscellaneous compounds in EU PDO Erzincan Tulum cheeses ripened in different package materials.
