# Supplementary figures and images for: A Sustainable and Alternative Packaging Approach for EU PDO Erzincan Tulum Cheese Affecting Food Safety, Proteolysis, Lipolysis, and Volatilome in Cheese: Sausage Casing
Source: J Food Sci. 2026 May 12;91:e71111. doi: 10.1111/1750-3841.71111 (PMC13162753; doi:10.1111/1750-3841.71111)

## Slide 1
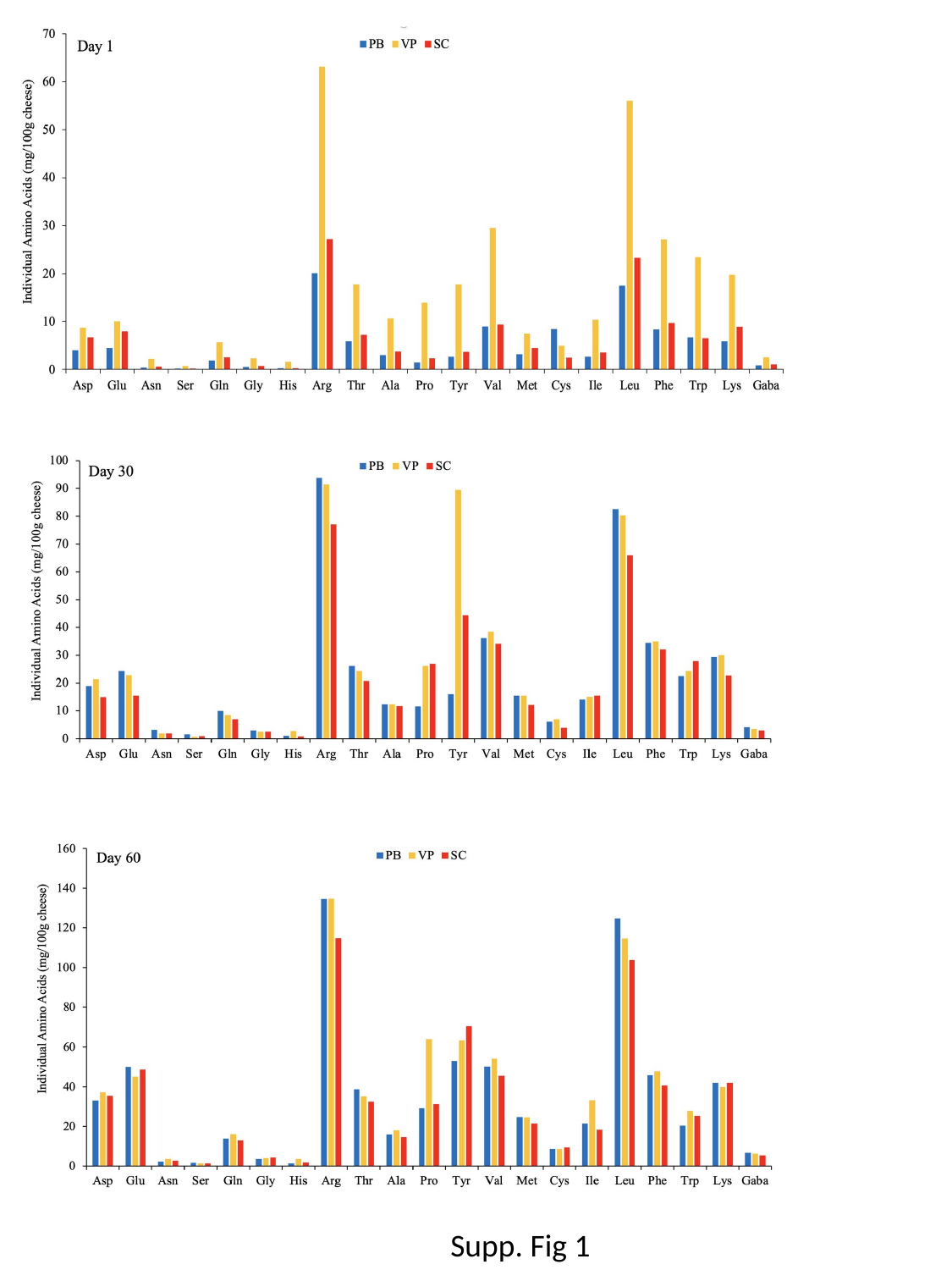

Supp. Fig 1

## Slide 2
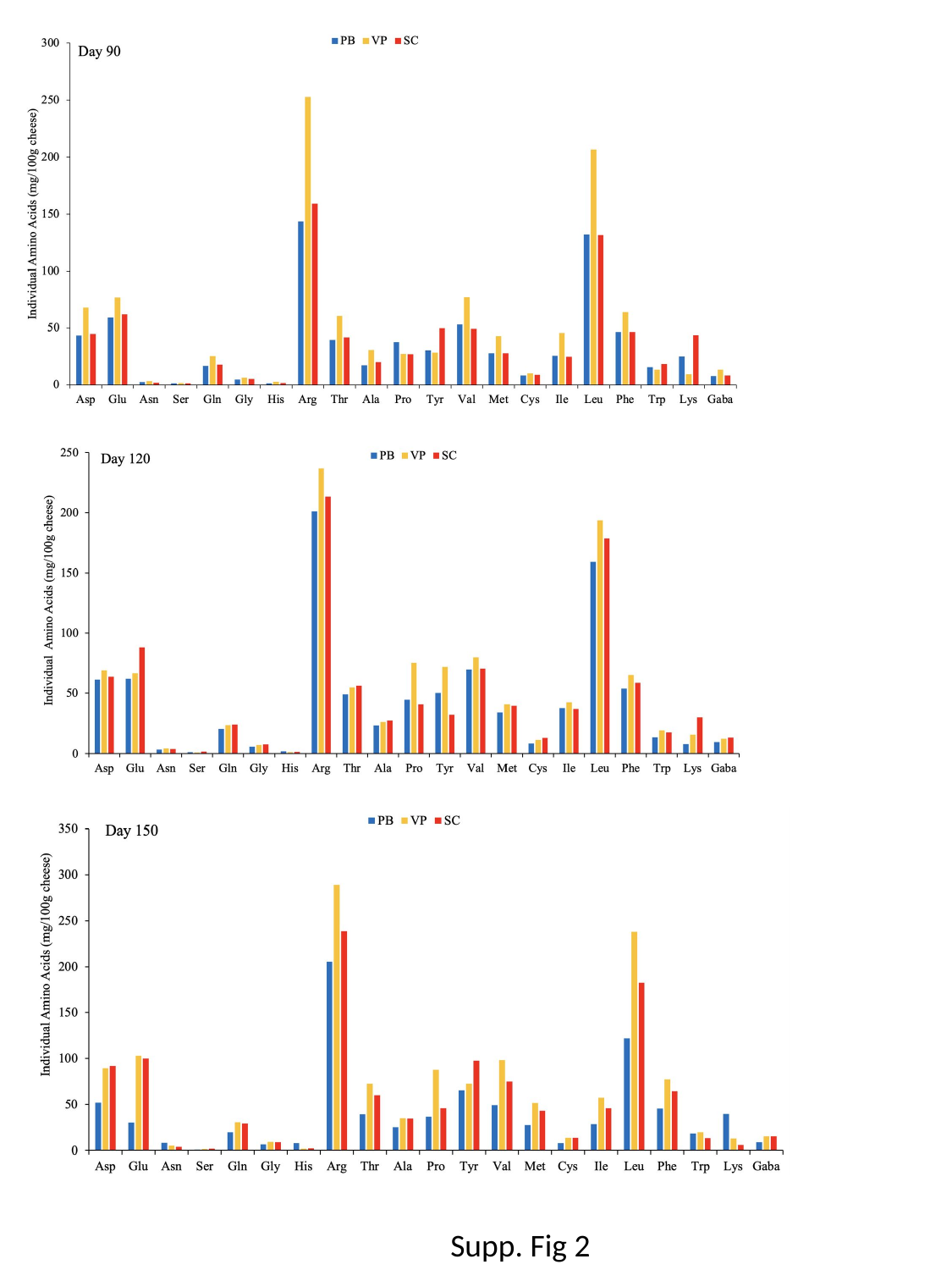

Supp. Fig 2

Supplement: Supplementary file 1 — Supplementary Figures: jfds71111‐sup‐0001‐Suppl. Figures R1.pptx [file JFDS-91-0-s002.pptx]
