# Supplementary material for: A Sustainable and Alternative Packaging Approach for EU PDO Erzincan Tulum Cheese Affecting Food Safety, Proteolysis, Lipolysis, and Volatilome in Cheese: Sausage Casing
Source: J Food Sci. 2026 May 12;91:e71111. doi: 10.1111/1750-3841.71111 (PMC13162753; doi:10.1111/1750-3841.71111)
Supplement: Supplementary file 2 — Table S1 Levels of individual free amino acids (mg/100 g cheese) in EU PDO Erzincan Tulum cheeses ripened in different package materials. Table S2 Alcohols in EU PDO Erzincan Tulum cheeses ripened in different package materials. Table S3 Esters in EU PDO Erzincan Tulum cheeses ripened in different package materials. Table S4 Acids in EU PDO Erzincan Tulum cheeses ripened in different package materials. Table S5 Aldehydes and ketones in EU PDO Erzincan Tulum cheeses ripened in different package materials. Table S6 Hydrocarbons in EU PDO Erzincan Tulum cheeses ripened in different package materials. Table S7 Terpenes in EU PDO Erzincan Tulum cheeses ripened in different package materials. Table S8 Miscellaneous compounds in EU PDO Erzincan Tulum cheeses ripened in different package materials. [file JFDS-91-0-s001.docx]

Supplementary Table S1. Levels of individual free amino acids (mg/100 g cheese) in EU PDO Erzincan Tulum cheeses ripened in different package materials

| **Amino acids** | **Cheeses** | **Day 1** | **Day 30** | **Day 60** | **Day 90** | **Day 120** | **Day 150** | ***P_day_*** |
| --- | --- | --- | --- | --- | --- | --- | --- | --- |
| **Asp** | **PB** | 4.03±0.03 | 19.02±0.05 | 33.05±0.01 | 43.48±0.20 | 61.33±0.01 | 51.84±0.01 | *** |
|  | **SC** | 6.69±0.14 | 14.92±0.37 | 35.42±0.07 | 44.76±0.18 | 63.89±0.02 | 91.76±0.12 | *** |
|  | **VP** | 8.68±0.12 | 21.49±0.12 | 37.25±0.03 | 67.71±0.48 | 69.05±0.09 | 89.55±0.16 | *** |
| ***P_sample_*** |  | ** | * | * | * | * | ** |  |
| **Glu** | **PB** | 4.53±0.06 | 24.25±0.07 | 49.95±0.02 | 59.31±0.11 | 62.13±0.11 | 30.34±0.07 | *** |
|  | **SC** | 8.01±0.44 | 15.49±0.77 | 48.64±0.07 | 62.00±0.16 | 87.93±0.09 | 100.05±0.12 | *** |
|  | **VP** | 10.02±0.03 | 22.79±0.16 | 45.11±0.23 | 76.77±0.14 | 66.80±0.17 | 102.89±0.17 | *** |
| ***P_sample_*** |  | ** | * | * | ** | ** | *** |  |
| **Asn** | **PB** | 0.41±0.04 | 3.18±0.00 | 2.31±0.11 | 2.48±0.02 | 3.43±0.01 | 8.27±0.12 | ** |
|  | **SC** | 0.59±0.00 | 1.92±0.18 | 2.74±0.02 | 1.80±0.02 | 3.84±0.29 | 3.75±0.24 | ** |
|  | **VP** | 2.18±0.05 | 1.92±0.08 | 3.54±0.29 | 3.22±0.02 | 4.41±0.25 | 5.20±0.20 | * |
| ***P_sample_*** |  | * | ** | * | * | * | ** |  |
| **Ser** | **PB** | 0.21±0.01 | 1.56±0.05 | 1.69±0.10 | 1.30±0.06 | 1.27±0.02 | 0.87±0.19 | NS |
|  | **SC** | 0.21±0.00 | 0.99±0.12 | 1.30±0.04 | 1.42±0.05 | 1.79±0.23 | 1.75±0.18 | ** |
|  | **VP** | 0.74±0.08 | 0.68±0.10 | 1.35±0.19 | 1.59±0.01 | 1.10±0.22 | 1.66±0.15 | ** |
| ***P_sample_*** |  | * | NS | NS | NS | NS | ** |  |
| **Gln** | **PB** | 1.85±0.06 | 9.95±0.01 | 13.80±0.04 | 16.59±0.09 | 20.35±0.05 | 19.91±0.19 | *** |
|  | **SC** | 2.54±0.01 | 6.87±0.31 | 13.01±0.02 | 17.70±0.10 | 24.04±0.02 | 29.40±0.09 | *** |
|  | **VP** | 5.70±0.02 | 8.48±0.09 | 16.05±0.10 | 25.24±0.03 | 23.65±0.06 | 30.59±0.04 | *** |
| ***P_sample_*** |  | ** | * | * | ** | * | ** |  |
| **Gly** | **PB** | 0.56±0.04 | 3.01±0.01 | 3.63±0.02 | 4.70±0.15 | 5.81±0.01 | 6.74±0.05 | *** |
|  | **SC** | 0.78±0.02 | 2.60±0.14 | 4.39±0.22 | 5.37±0.07 | 7.77±0.08 | 9.11±0.13 | *** |
|  | **VP** | 2.31±0.01 | 2.62±0.12 | 4.03±0.12 | 6.47±0.85 | 7.30±0.03 | 9.30±0.07 | *** |
| ***P_sample_*** |  | ** | *** | *** | *** | *** | *** |  |
| **His** | **PB** | 0.24±0.05 | 1.01±0.07 | 1.42±0.09 | 1.28±0.11 | 2.00±0.02 | 8.02±0.04 | *** |
|  | **SC** | 0.25±0.03 | 0.89±0.30 | 1.73±0.66 | 1.52±0.17 | 1.45±0.13 | 2.33±0.36 | ** |
|  | **VP** | 1.55±0.05 | 2.79±0.01 | 3.58±0.02 | 2.68±1.82 | 1.26±0.13 | 1.78±0.05 | * |
| ***P_sample_*** |  | * | *** | *** | NS | *** | *** |  |
| **Arg** | **PB** | 20.09±0.03 | 93.80±0.17 | 134.52±0.02 | 143.49±0.21 | 201.00±0.74 | 205.57±0.18 | *** |
|  | **SC** | 27.12±0.00 | 76.99±0.43 | 114.75±0.16 | 159.02±0.06 | 213.40±0.46 | 238.33±0.24 | *** |
|  | **VP** | 63.08±0.60 | 91.39±0.04 | 134.77±0.02 | 252.82±0.19 | 236.70±0.36 | 288.85±0.39 | *** |
| ***P_sample_*** |  | *** | *** | *** | *** | *** | *** |  |
| **Thr** | **PB** | 5.87±0.05 | 26.16±0.04 | 38.73±0.05 | 39.41±0.03 | 48.87±0.03 | 39.50±0.07 | *** |
|  | **SC** | 7.19±0.01 | 20.78±0.20 | 32.53±0.02 | 41.74±0.00 | 56.26±0.23 | 60.13±0.17 | *** |
|  | **VP** | 17.75±0.18 | 24.33±0.07 | 35.20±0.02 | 60.62±0.04 | 54.71±0.09 | 72.43±0.20 | *** |
| ***P_sample_*** |  | *** | *** | *** | *** | *** | *** |  |
| **Ala** | **PB** | 3.01±0.03 | 12.30±0.07 | 15.95±0.15 | 17.16±0.02 | 23.18±0.05 | 25.13±0.21 | *** |
|  | **SC** | 3.72±0.07 | 11.70±0.16 | 14.65±0.03 | 20.04±0.00 | 27.48±0.07 | 34.68±0.06 | *** |
|  | **VP** | 10.66±0.11 | 12.38±0.00 | 18.08±0.00 | 30.29±0.11 | 26.12±0.06 | 35.27±0.04 | *** |
| ***P_sample_*** |  | ** | *** | *** | *** | *** | *** |  |
| **Pro** | **PB** | 1.44±0.01 | 11.58±0.05 | 29.25±0.49 | 37.52±0.34 | 44.78±0.16 | 36.94±0.44 | *** |
|  | **SC** | 2.35±0.02 | 26.87±0.07 | 31.28±0.11 | 27.06±0.23 | 41.04±0.53 | 45.81±0.12 | *** |
|  | **VP** | 13.89±0.13 | 26.16±0.01 | 64.07±0.04 | 27.36±0.07 | 75.40±0.13 | 88.04±0.00 | *** |
| ***P_sample_*** |  | *** | *** | *** | *** | *** | *** |  |
| **Tyr** | **PB** | 2.71±0.11 | 16.01±0.05 | 53.05±0.02 | 30.23±0.07 | 50.16±0.00 | 65.55±0.13 | *** |
|  | **SC** | 3.66±0.05 | 44.38±0.05 | 70.62±0.05 | 49.60±0.10 | 32.18±3.71 | 97.43±2.27 | *** |
|  | **VP** | 17.74±0.01 | 89.47±0.16 | 63.36±0.03 | 28.34±1.23 | 72.06±0.01 | 72.62±0.07 | *** |
| ***P_sample_*** |  | ** | *** | *** | *** | *** | *** |  |
| **Val** | **PB** | 8.95±0.07 | 36.18±0.11 | 50.19±0.13 | 52.90±0.04 | 69.93±0.12 | 49.32±0.15 | *** |
|  | **SC** | 9.41±0.05 | 34.14±0.06 | 45.59±0.02 | 49.00±0.07 | 70.67±0.91 | 74.95±0.60 | *** |
|  | **VP** | 29.52±0.06 | 38.36±0.10 | 54.16±0.07 | 77.05±0.16 | 80.05±0.12 | 98.19±0.13 | *** |
| ***P_sample_*** |  | * | *** | *** | *** | *** | *** |  |
| **Met** | **PB** | 3.20±0.02 | 15.55±0.02 | 24.77±0.05 | 27.82±0.10 | 34.14±0.01 | 27.64±0.05 | *** |
|  | **SC** | 4.51±0.00 | 12.11±0.05 | 21.45±0.03 | 27.85±0.06 | 39.65±0.54 | 43.33±0.30 | *** |
|  | **VP** | 7.51±0.07 | 15.55±0.09 | 24.52±0.00 | 42.96±0.08 | 40.96±0.07 | 51.39±0.13 | *** |
| ***P_sample_*** |  | *** | *** | *** | *** | *** | *** |  |
| **Cys** | **PB** | 8.42±0.00 | 6.08±0.00 | 8.68±0.04 | 8.35±0.01 | 8.43±0.03 | 7.90±0.06 | *** |
|  | **SC** | 2.49±0.03 | 3.97±0.02 | 9.37±0.01 | 8.94±0.03 | 12.95±0.08 | 13.71±0.02 | *** |
|  | **VP** | 4.91±0.00 | 6.95±0.01 | 8.63±0.02 | 10.38±0.14 | 11.20±0.05 | 13.73±0.09 | *** |
| ***P_sample_*** |  | *** | *** | *** | *** | *** | *** |  |
| **Ile** | **PB** | 2.68±0.01 | 14.04±0.00 | 21.46±0.01 | 25.70±0.00 | 37.79±0.02 | 28.60±0.08 | *** |
|  | **SC** | 3.56±0.04 | 15.51±0.41 | 18.33±0.20 | 24.66±0.11 | 37.19±0.01 | 45.61±0.26 | *** |
|  | **VP** | 10.33±0.04 | 15.05±0.09 | 33.25±0.25 | 45.67±0.06 | 42.78±0.06 | 57.46±0.12 | *** |
| ***P_sample_*** |  | *** | *** | *** | *** | *** | *** |  |
| **Leu** | **PB** | 17.50±0.03 | 82.52±0.16 | 124.81±0.54 | 132.16±0.38 | 158.96±0.01 | 121.91±0.74 | *** |
|  | **SC** | 23.29±0.07 | 65.98±0.03 | 103.68±0.05 | 131.58±0.02 | 178.75±0.19 | 182.47±0.19 | *** |
|  | **VP** | 56.07±0.09 | 80.33±0.35 | 114.63±0.04 | 206.76±9.28 | 193.36±2.33 | 237.65±0.04 | *** |
| ***P_sample_*** |  | *** | *** | *** | *** | *** | *** |  |
| **Phe** | **PB** | 8.33±0.12 | 34.52±0.07 | 45.78±0.32 | 46.43±0.03 | 53.85±0.19 | 45.24±0.11 | *** |
|  | **SC** | 9.74±0.05 | 32.08±0.34 | 40.57±0.37 | 46.64±0.09 | 58.58±0.01 | 64.51±0.14 | *** |
|  | **VP** | 27.05±0.02 | 35.02±0.15 | 47.74±0.40 | 63.97±0.18 | 65.18±0.06 | 77.24±0.41 | *** |
| ***P_sample_*** |  | *** | *** | *** | *** | *** | *** |  |
| **Trp** | **PB** | 6.66±0.02 | 22.42±0.06 | 20.35±0.13 | 15.43±0.02 | 13.25±0.08 | 18.76±0.07 | *** |
|  | **SC** | 6.53±0.04 | 27.92±0.03 | 25.28±0.15 | 18.31±0.09 | 17.39±0.05 | 13.44±0.04 | *** |
|  | **VP** | 23.47±0.10 | 24.27±0.27 | 27.80±0.02 | 13.09±0.06 | 19.16±0.04 | 20.03±0.13 | *** |
| ***P_sample_*** |  | * | *** | *** | *** | *** | *** |  |
| **Lys** | **PB** | 5.89±0.03 | 29.37±0.09 | 42.02±0.02 | 24.94±0.22 | 7.96±0.05 | 40.05±0.06 | *** |
|  | **SC** | 8.90±0.03 | 22.64±0.01 | 42.03±0.00 | 43.64±0.10 | 30.15±0.02 | 6.06±0.02 | *** |
|  | **VP** | 19.78±0.61 | 30.01±0.04 | 39.96±0.05 | 9.51±0.02 | 15.60±0.07 | 13.20±0.01 | *** |
| ***P_sample_*** |  | *** | *** | *** | *** | *** | *** |  |
| **GABA** | **PB** | 0.87±0.01 | 4.22±0.10 | 6.73±0.01 | 7.80±0.04 | 9.75±0.05 | 8.92±0.18 | *** |
|  | **SC** | 1.12±0.00 | 3.00±0.13 | 5.42±0.06 | 8.32±0.05 | 13.10±0.23 | 15.70±1.45 | *** |
|  | **VP** | 2.59±0.10 | 3.54±0.07 | 6.24±0.02 | 13.20±0.21 | 12.07±1.02 | 15.39±0.11 | *** |
| ***P_sample_*** |  | *** | *** | *** | *** | *** | *** |  |

NS: Not significant. *P_day_* and *P_sample_* imply the effect of ripening time and sample type on the levels of individual free amino acids, respectively; PB: Plastic barrel; VP: Vacuum package; SC: Sausage casing; *: *P*<0.05; **: *P*<0.01; ***: *P*<0.001

Supplementary Table S2. Alcohols in EU PDO Erzincan Tulum cheeses ripened in different package materials

| **Volatile compounds** | **Cheeses** | **Day 1** | **Day 30** | **Day 60** | ***P_day_*** |
| --- | --- | --- | --- | --- | --- |
| ***Alcohols*** |  |  |  |  |  |
| Ethanol | **PB** | 92.74±1.69 | 81.47±5.30 | 79.54±1.31 | * |
|  | **SC** | 107.28±4.54 | 89.23±1.32 | 82.40±12.30 | * |
|  | **VP** | 89.84±7.62 | 90.92±4.00 | 88.47±4.75 | NS |
| ***P_sample_*** |  | NS | NS | NS |  |
| 1-Propanol | **PB** | 0.39±0.04 | 0.11±0.00 | N.D. | NS |
|  | **SC** | 0.38±0.12 | N.D. | 0.15±0.00 |  |
|  | **VP** | 0.35±0.06 | N.D. | 0.30±0.00 | NS |
| ***P_sample_*** |  | NS | NS | NS |  |
| 2-Methyl-1-propanol | **PB** | 31.27±4.42 | 21.96±3.41 | 8.58±0.02 | * |
|  | **SC** | 43.72±4.29 | 6.02±5.87 | 0.88±0.19 | ** |
|  | **VP** | 42.94±3.69 | 11.98±2.89 | 1.04±0.18 | ** |
| ***P_sample_*** |  | * | * | * |  |
| 3-Methyl-1-butanol | **PB** | 134.77±6.68 | 45.26±1.41 | 18.99±0.46 | ** |
|  | **SC** | 153.32±6.18 | 19.89±1.90 | 9.93±2.73 | ** |
|  | **VP** | 160.41±7.32 | 30.22±0.12 | 14.05±0.58 | *** |
| ***P_sample_*** |  | NS | * | * |  |
| 2-Methyl-1-butanol | **PB** | 14.33±1.09 | 8.29±0.93 | 3.31±0.01 | * |
|  | **SC** | 15.91±0.09 | 3.14±0.39 | 1.35±0.49 | * |
|  | **VP** | 13.07±1.07 | 3.95±0.45 | 2.27±0.00 | * |
| ***P_sample_*** |  | NS | * | NS |  |
| 2.3-Butanediol | **PB** | 1.18±0.35 | 6.99±1.80 | 5.59±0.16 | * |
|  | **SC** | 3.00±0.79 | 14.16±1.96 | 4.49±0.10 | * |
|  | **VP** | 3.03±0.08 | 4.05±1.60 | 2.98±0.28 | NS |
| ***P_sample_*** |  | * | * | * |  |
| 3-Methyl-2-hexanol | **PB** | N.D. | 4.65±0.00 | 3.03±0.00 | * |
|  | **SC** | 1.07±0.00 | 3.98±0.00 | N.D. | * |
|  | **VP** | 1.27±0.00 | 2.58±1.25 | N.D. | * |
| ***P_sample_*** |  | * | NS | * |  |
| 3-(2.2-Dimethylpropoxy)-2-butanol | **PB** | N.D. | 0.28±0.00 | 1.81±0.00 | * |
|  | **SC** | N.D. | 0.87±0.00 | 1.68±0.18 | * |
|  | **VP** | N.D. | 0.43±0.00 | 2.03±0.42 | * |
| ***P_sample_*** |  | NS | NS | NS |  |

ND: Not detected. NS: Not significant. *P_day_* and *P_sample_* imply the effect of ripening time and sample type on the levels of alcohols, respectively; PB: Plastic barrel; VP: Vacuum package; SC: Sausage casing; *: *P*<0.05; **: *P*<0.01; ***: *P*<0.001

Supplementary Table S3. Esters in EU PDO Erzincan Tulum cheeses ripened in different package materials

| **Volatile compounds** | **Cheeses** | **Day 1** | **Day 30** | **Day 60** | ***P_day_*** |
| --- | --- | --- | --- | --- | --- |
| ***Esters*** |  |  |  |  |  |
| Methyl acetate | **PB** | 0.75±0.06 | 1.07±0.10 | 0.82±0.33 | NS |
|  | **SC** | 0.35±0.03 | 0.84±0.15 | 0.96±1.04 | NS |
|  | **VP** | 0.47±0.04 | 0.95±0.12 | 0.88±0.14 | NS |
| ***P_sample_*** |  | NS | NS | NS |  |
| Ethyl acetate | **PB** | 150.62±6.74 | 135.05±12.36 | 98.41±10.87 | * |
|  | **SC** | 205.96±14.00 | 111.07±0.81 | 141.45±7.99 | ** |
|  | **VP** | 223.88±10.47 | 109.55±0.97 | 103.19±26.27 | * |
| ***P_sample_*** |  | * | NS | NS |  |
| Ethyl propanoate | **PB** | 1.48±0.16 | 0.49±0.01 | N.D. | NS |
|  | **SC** | 1.69±0.16 | 0.36±0.01 | 0.71±0.25 | * |
|  | **VP** | 2.22±0.20 | 0.62±0.37 | 0.61±0.33 | * |
| ***P_sample_*** |  | * | NS | NS |  |
| Propyl acetate | **PB** | 2.94±0.44 | 0.91±0.07 | 3.58±0.00 | * |
|  | **SC** | 3.58±0.30 | 0.66±0.05 | 1.24±0.26 | * |
|  | **VP** | 4.86±0.59 | 0.90±0.14 | 1.16±0.43 | ** |
| ***P_sample_*** |  | NS | NS | ** |  |
| Methyl butanoate | **PB** | N.D. | 0.58±0.10 | N.D. | * |
|  | **SC** | N.D. | 0.97±0.00 | 1.46±0.00 | ** |
|  | **VP** | N.D. | 0.52±0.07 | 0.65±0.00 | * |
| ***P_sample_*** |  | NS | * | NS |  |
| Ethyl-2-methyl-propanoate | **PB** | 0.78±0.33 | 0.47±0.00 | 0.78±0.06 | NS |
|  | **SC** | 0.52±0.28 | 0.25±0.00 | 0.64±0.08 | NS |
|  | **VP** | 0.80±0.19 | 0.38±0.00 | 0.93±0.00 | * |
| ***P_sample_*** |  | NS | NS | * |  |
| 2-Methylpropyl acetate | **PB** | 7.38±0.56 | 2.88±0.26 | 2.92±0.60 | ** |
|  | **SC** | 8.08±0.26 | 3.93±0.10 | 4.81±1.52 | ** |
|  | **VP** | 12.91±1.85 | 4.72±2.44 | 3.44±0.53 | ** |
| ***P_sample_*** |  | * | NS | NS |  |
| Ethyl butanoate | **PB** | 14.05±0.17 | 22.94±0.30 | 41.39±8.33 | ** |
|  | **SC** | 29.08±0.00 | 27.01±6.68 | 74.75±9.35 | ** |
|  | **VP** | 10.66±0.22 | 27.12±4.72 | 49.30±2.37 | *** |
| ***P_sample_*** |  | *** | * | * |  |
| Ethyl lactate | **PB** | 6.52±2.29 | N.D. | N.D. | ** |
|  | **SC** | 8.37±0.57 | 25.46±0.00 | 3.12±0.00 | ** |
|  | **VP** | 4.28±0.00 | 27.19±0.00 | 22.56±0.00 | *** |
| ***P_sample_*** |  | NS | ** | ** |  |
| 3-Methyl-1-butyl acetate | **PB** | 36.40±3.98 | 19.19±0.04 | 22.27±0.00 | ** |
|  | **SC** | 36.29±2.09 | 34.65±0.00 | 27.55±0.00 | * |
|  | **VP** | 68.07±18.84 | 35.42±9.35 | 21.44±0.07 | *** |
| ***P_sample_*** |  | ** | ** | NS |  |
| 2.3-Butanedioldiacetate | **PB** | 2.03±0.32 | 0.44±0.05 | 2.60±0.00 | * |
|  | **SC** | 2.16±0.15 | 1.33±0.49 | 0.50±0.00 | * |
|  | **VP** | 1.23±0.00 | 1.32±0.12 | 0.36±0.00 | * |
| ***P_sample_*** |  | NS | * | * |  |
| Methyl-2-hydroxy-4-methyl-pentanoate | **PB** | 0.56±0.05 | 0.39±0.13 | 9.60±0.86 | ** |
|  | **SC** | N.D. | N.D. | N.D. | NS |
|  | **VP** | 0.93±0.22 | 0.74±0.00 | 1.38±0.80 | * |
| ***P_sample_*** |  | NS | ** | ** |  |
| Ethyl hexanoate | **PB** | 3.20±0.26 | 31.44±2.14 | 70.13±0.00 | * |
|  | **SC** | 2.21±0.07 | 35.90±3.11 | 60.08±9.87 | ** |
|  | **VP** | 3.05±0.23 | 27.59±0.00 | 89.93±14.92 | *** |
| ***P_sample_*** |  | * | NS | * |  |
| 3-Methyl butyl butanoate | **PB** | 0.20±0.01 | 1.09±0.29 | 3.28±0.21 | ** |
|  | **SC** | 0.14±0.00 | 1.51±0.14 | 2.77±0.00 | * |
|  | **VP** | 2.20±0.79 | 4.82±0.52 | 4.83±2.41 | * |
| ***P_sample_*** |  | * | ** | * |  |
| Ethyl octanoate | **PB** | 0.38±0.10 | 2.28±0.60 | 6.52±0.00 | * |
|  | **SC** | 0.18±0.00 | 3.71±0.20 | 5.76±0.75 | ** |
|  | **VP** | N.D. | 2.96±1.05 | 5.13±1.96 | * |
| ***P_sample_*** |  | NS | NS | NS |  |
| 2-Phenyl ethyl acetate | **PB** | 1.08±0.12 | 2.02±0.32 | 3.16±0.00 | NS |
|  | **SC** | N.D. | 2.80±0.42 | 1.96±0.14 | * |
|  | **VP** | 1.41±0.00 | 2.81±0.85 | 2.08±0.74 | * |
| ***P_sample_*** |  | * | NS | NS |  |
| Ethyl decanoate | **PB** | N.D. | 0.42±0.00 | 1.55±0.33 | * |
|  | **SC** | N.D. | 0.99±0.00 | 1.45±0.08 | * |
|  | **VP** | N.D. | 0.82±0.38 | 1.37±0.42 | * |
| ***P_sample_*** |  | NS | NS | NS |  |

ND: Not detected. NS: Not significant. NS: Not significant. *P_day_* and *P_sample_* imply the effect of ripening time and sample type on the levels of esters, respectively; PB: Plastic barrel; VP: Vacuum package; SC: Sausage casing; *: *P*<0.05; **: *P*<0.01; ***: *P*<0.001

Supplementary Table S4. Acids in EU PDO Erzincan Tulum cheeses ripened in different package materials

| **Volatile compounds** | **Cheeses** | **Day 1** | **Day 30** | **Day 60** | ***P_day_*** |
| --- | --- | --- | --- | --- | --- |
| ***Acids*** |  |  |  |  |  |
| Acetic acid | **PB** | 45.60±2.72 | 45.50±5.10 | 68.02±2.85 | ** |
|  | **SC** | 27.70±4.07 | 75.58±15.84 | 55.53±15.62 | * |
|  | **VP** | 27.77±12.57 | 52.26±17.87 | 54.11±4.09 | * |
| ***P_sample_*** |  | * | * | * |  |
| Butanoic acid | **PB** | 3.48±0.61 | 31.84±1.21 | 132.15±7.38 | ** |
|  | **SC** | N.D. | 67.16±3.73 | 137.06±18.44 | * |
|  | **VP** | 1.78±0.91 | 30.04±6.67 | 88.49±3.89 | * |
| ***P_sample_*** |  | * | ** | * |  |
| 3-Methyl butanoic acid | **PB** | 3.20±0.43 | 6.05±1.35 | 11.68±0.00 | * |
|  | **SC** | 5.92±0.13 | 12.47±1.38 | 5.23±0.00 | * |
|  | **VP** | 8.46±0.97 | 11.31±0.36 | 7.89±2.38 | NS |
| ***P_sample_*** |  | * | * | * |  |
| 2-Methyl butanoic acid | **PB** | 0.48±0.21 | 1.14±0.00 | N.D. | NS |
|  | **SC** | 0.71±0.07 | N.D. | N.D. | NS |
|  | **VP** | 1.50±0.14 | 4.22±0.00 | 1.34±0.00 | * |
| ***P_sample_*** |  | NS | * | * |  |
| Hexanoic acid | **PB** | 1.72±0.58 | 6.32±0.00 | 93.15±12.82 | *** |
|  | **SC** | 1.17±0.02 | 23.50±2.68 | 61.12±4.28 | ** |
|  | **VP** | 1.02±0.31 | 7.43±1.10 | 44.56±0.33 | *** |
| ***P_sample_*** |  | NS | ** | * |  |
| Octanoic Acid | **PB** | N.D. | N.D. | 8.17±1.46 | * |
|  | **SC** | N.D. | 2.30±0.16 | 3.67±0.14 | NS |
|  | **VP** | N.D. | N.D. | 3.80±0.00 | ** |
| ***P_sample_*** |  | NS | NS | * |  |

ND: Not detected. NS: Not significant. *P_day_* and *P_sample_* imply the effect of ripening time and sample type on the levels of acids respectively;; PB: Plastic barrel; VP: Vacuum package; SC: Sausage casing; *: *P*<0.05; **: *P*<0.01; ***: *P*<0.001

Supplementary Table S5. Aldehydes and ketones in EU PDO Erzincan Tulum cheeses ripened in different package materials

| **Volatile compounds** | **Cheeses** | **Day 1** | **Day 30** | **Day 60** | ***P_day_*** |
| --- | --- | --- | --- | --- | --- |
| ***Aldehydes and Ketones*** |  |  |  |  |  |
| Acetaldehyde | **PB** | 2.53±0.29 | 1.55±0.00 | 1.61±1.01 | NS |
|  | **SC** | 3.09±0.00 | 0.07±0.02 | 1.97±0.21 | * |
|  | **VP** | 3.75±0.75 | N.D. | 3.27±0.26 | NS |
| ***P_sample_*** |  | NS | NS | NS |  |
| 3-Methyl-butanal | **PB** | 1.92±0.00 | 2.28±0.00 | N.D. | NS |
|  | **SC** | 1.59±0.66 | 1.25±0.00 | 1.92±0.00 | NS |
|  | **VP** | 2.17±0.00 | N.D. | N.D. | NS |
| ***P_sample_*** |  | NS | NS | NS |  |
| Nonanal | **PB** | 0.27±0.00 | 0.90±0.78 | N.D. | NS |
|  | **SC** | N.D. | N.D. | N.D. | NS |
|  | **VP** | 0.29±0.00 | 0.87±0.24 | N.D. | NS |
| ***P_sample_*** |  | NS | NS | NS |  |
| Diacetyl | **PB** | 0.28±0.04 | 0.65±0.49 | 9.01±1.56 | * |
|  | **SC** | N.D. | 0.29±0.00 | 0.23±0.22 | NS |
|  | **VP** | 0.57±0.28 | 0.21±0.00 | 1.28±0.25 | NS |
| ***P_sample_*** |  | NS | NS | ** |  |
| 2-Pentanone | **PB** | 0.14±0.04 | 0.56±0.45 | 0.94±0.08 | NS |
|  | **SC** | 0.20±0.03 | 0.32±0.04 | 0.27±0.20 | NS |
|  | **VP** | 0.30±0.03 | 0.26±0.00 | 0.26±0.01 | * |
| ***P_sample_*** |  | * | * | ** |  |
| Acetoin | **PB** | 0.82±0.00 | 0.32±0.00 | 27.69±3.19 | *** |
|  | **SC** | N.D. | 1.41±0.00 | 0.94±0.15 | * |
|  | **VP** | N.D. | 0.51±0.00 | 2.29±0.49 | ** |
| ***P_sample_*** |  | NS | NS | *** |  |

ND: Not detected. NS: Not significant. *P_day_* and *P_sample_* imply the effect of ripening time and sample type on the levels of aldehydes and ketones, respectively; PB: Plastic barrel; VP: Vacuum package; SC: Sausage casing; *: *P*<0.05; **: *P*<0.01; ***: *P*<0.001

Supplementary Table S6. Hydrocarbons in EU PDO Erzincan Tulum cheeses ripened in different package materials

| **Volatile compounds** | **Cheeses** | **Day 1** | **Day 30** | **Day 60** | ***P_day_*** |
| --- | --- | --- | --- | --- | --- |
| ***Hydrocarbons*** |  |  |  |  |  |
| 2.3-Dimethyl-butane | **PB** | 3.41±0.06 | 7.90±2.42 | 9.12±0.68 | * |
|  | **SC** | 1.00±0.53 | 7.49±1.68 | 3.65±0.99 | * |
|  | **VP** | 0.91±0.27 | 10.32±0.44 | 5.59±0.76 | NS |
| ***P_sample_*** |  | ** | * | * |  |
| 3-Methyl-pentane | **PB** | 1.21±0.04 | 3.38±1.09 | 4.25±0.74 | * |
|  | **SC** | 0.32±0.26 | 2.98±0.96 | 1.09±0.36 | * |
|  | **VP** | N.D. | 4.69±0.87 | 2.15±0.42 | NS |
| ***P_sample_*** |  | NS | NS | * |  |
| Methyl-cyclopentane | **PB** | N.D. | N.D. | 13.84±0.33 | *** |
|  | **SC** | N.D. | 11.24±3.11 | 16.82±0.00 | ** |
|  | **VP** | N.D. | 16.32±0.00 | 12.73±0.00 | ** |
| ***P_sample_*** |  | NS | ** | NS |  |
| Cyclohexane | **PB** | 0.83±0.12 | 1.97±0.00 | 2.13±0.33 | NS |
|  | **SC** | 0.55±0.00 | 2.33±0.00 | 2.47±0.00 | * |
|  | **VP** | 0.70±0.14 | 2.61±0.00 | 1.77±0.62 | * |
| ***P_sample_*** |  | NS | NS | NS |  |
| Heptane | **PB** | 1.16±0.12 | 1.59±0.48 | 0.88±0.24 | NS |
|  | **SC** | 4.52±0.74 | 1.86±0.20 | 1.21±0.41 | * |
|  | **VP** | 3.11±0.51 | 1.51±0.22 | 1.04±0.19 | NS |
| ***P_sample_*** |  | * | NS | NS |  |
| 1-Octene | **PB** | N.D. | N.D. | N.D. | NS |
|  | **SC** | 1.03±0.45 | 1.15±0.75 | 4.63±0.00 | * |
|  | **VP** | N.D. | N.D. | N.D. | NS |
| ***P_sample_*** |  | * | * | * |  |
| m-Xylene | **PB** | 0.42±0.14 | 7.55±0.18 | 35.19±0.00 | ** |
|  | **SC** | 0.38±0.00 | 31.37±0.00 | 29.52±0.00 | *** |
|  | **VP** | 0.48±0.29 | 8.56±0.00 | 0.18±0.00 | * |
| ***P_sample_*** |  | NS | ** | *** |  |
| o-Xylene | **PB** | N.D. | 1.67±0.00 | 7.23±0.00 | ** |
|  | **SC** | N.D. | N.D. | 5.07±0.00 | *** |
|  | **VP** | 0.58±0.00 | 1.67±0.00 | 4.96±0.00 | *** |
| ***P_sample_*** |  | ** | * | * |  |
| 2.5-Dimethyl-1.6-octadiene | **PB** | 0.71±0.10 | 0.80±0.10 | 1.61±0.30 | NS |
|  | **SC** | 0.77±0.03 | 0.95±0.07 | 0.67±0.16 | NS |
|  | **VP** | N.D. | N.D. | N.D. | NS |
| ***P_sample_*** |  | *** | ** | ** |  |
| 2.2.4-Trimethyl-heptane | **PB** | N.D. | N.D. | N.D. | NS |
|  | **SC** | N.D. | N.D. | N.D. | NS |
|  | **VP** | 5.46±2.22 | 5.58±2.54 | 3.83±1.27 | * |
| ***P_sample_*** |  | * | * | * |  |
| 3.7-Dimethyl-2-octene | **PB** | 0.91±0.11 | 1.17±0.61 | 2.33±0.33 | ** |
|  | **SC** | 0.97±0.04 | 1.56±0.34 | 1.32±0.75 | * |
|  | **VP** | 1.49±0.51 | 1.18±0.41 | 1.01±0.21 | * |
| ***P_sample_*** |  | * | NS | NS |  |
| 2.2.3-Trimethyl-hexane | **PB** | N.D. | N.D. | N.D. | NS |
|  | **SC** | N.D. | N.D. | N.D. | NS |
|  | **VP** | 1.83±0.66 | 2.50±0.80 | 2.00±0.75 | * |
| ***P_sample_*** |  | * | * | * |  |
| 2.2-Dimethyl-undecane | **PB** | N.D. | N.D. | N.D. | NS |
|  | **SC** | N.D. | N.D. | N.D. | NS |
|  | **VP** | 2.45±0.50 | 3.51±0.00 | 1.10±0.00 | * |
| ***P_sample_*** |  | * | * | * |  |
| 2.2-Dimethyl-decane | **PB** | N.D. | N.D. | N.D. | NS |
|  | **SC** | 0.84±0.00 | 12.47±0.99 | 10.50±0.00 | ** |
|  | **VP** | 46.31±0.00 | 62.45±0.00 | 50.63±0.00 | * |
| ***P_sample_*** |  | *** | *** | *** |  |
| 2.2.3-Trimethyl-nonane | **PB** | N.D. | N.D. | N.D. | NS |
|  | **SC** | N.D. | N.D. | N.D. | NS |
|  | **VP** | 1.54±0.41 | 3.72±0.37 | 3.50±0.00 | * |
| ***P_sample_*** |  | * | * | * |  |
| 3.7-Dimethyl-nonane | **PB** | N.D. | N.D. | N.D. | NS |
|  | **SC** | 0.20±0.08 | N.D. | N.D. | NS |
|  | **VP** | 11.23±5.23 | 16.78±4.92 | 8.09±0.00 | ** |
| ***P_sample_*** |  | *** | *** | *** |  |
| 2.5-Dimethyl-undecane | **PB** | N.D. | N.D. | N.D. | NS |
|  | **SC** | N.D. | N.D. | N.D. | NS |
|  | **VP** | 3.09±1.45 | 2.29±0.00 | N.D. | * |
| ***P_sample_*** |  | ** | * | NS |  |
| 5.6-Dimethyl-decane | **PB** | N.D. | N.D. | N.D. | NS |
|  | **SC** | N.D. | N.D. | N.D. | NS |
|  | **VP** | 0.61±0.16 | 1.02±0.79 | 0.64±0.02 | * |
| ***P_sample_*** |  | * | * | * |  |
| 5-Ethyl-2.2.3-trimethyl-heptane | **PB** | N.D. | N.D. | N.D. | NS |
|  | **SC** | N.D. | N.D. | N.D. | NS |
|  | **VP** | 7.36±3.49 | 10.68±2.95 | 9.67±1.24 | * |
| ***P_sample_*** |  | *** | *** | *** |  |
| 2.2.5-Trimethyl-hexane | **PB** | N.D. | N.D. | N.D. | NS |
|  | **SC** | N.D. | N.D. | N.D. | NS |
|  | **VP** | 6.37±3.10 | 8.40±2.24 | 6.42±1.32 | * |
| ***P_sample_*** |  | *** | *** | *** |  |
| 9-Methyl-3-undecene | **PB** | N.D. | N.D. | N.D. | NS |
|  | **SC** | N.D. | N.D. | N.D. | NS |
|  | **VP** | 0.29±0.00 | 1.19±0.00 | 1.12±0.00 | NS |
| ***P_sample_*** |  | NS | NS | NS |  |

ND: Not detected. NS: Not significant. *P_day_* and *P_sample_* imply the effect of ripening time and sample type on the levels of hydrocarbons, respectively; PB: Plastic barrel; VP: Vacuum package; SC: Sausage casing; *: *P*<0.05; **: *P*<0.01; ***: *P*<0.001

Supplementary Table S7. Terpenes in EU PDO Erzincan Tulum cheeses ripened in different package materials

| **Volatile compounds** | **Cheeses** | **Day 1** | **Day 30** | **Day 60** | **P*_day_*** |
| --- | --- | --- | --- | --- | --- |
| ***Terpenes*** |  |  |  |  |  |
| 𝛼-Pinene | **PB** | 0.63±0.08 | 2.70±0.16 | 2.23±1.07 | NS |
|  | **SC** | 0.76±0.05 | 3.39±0.12 | 2.11±0.98 | * |
|  | **VP** | 0.48±0.05 | 1.94±0.65 | 1.47±0.50 | * |
| ***P_sample_*** |  | * | NS | NS |  |
| 𝛼-Citronellol | **PB** | N.D. | N.D. | N.D. | NS |
|  | **SC** | N.D. | N.D. | N.D. | NS |
|  | **VP** | 1.28±0.18 | 1.74±0.57 | 1.18±0.44 | * |
| ***P_sample_*** |  | *** | * | * |  |
| 𝛽-Ocimene | **PB** | 1.72±0.18 | 2.23±0.54 | N.D. | NS |
|  | **SC** | 1.94±0.03 | 3.53±0.24 | N.D. | NS |
|  | **VP** | 1.85±0.09 | 2.17±0.00 | N.D. | NS |
| ***P_sample_*** |  | NS | NS | NS |  |
| *p-Cymene* | **PB** | 0.40±0.00 | 0.72±0.00 | N.D. | NS |
|  | **SC** | 0.37±0.05 | N.D. | N.D. | NS |
|  | **VP** | 0.60±0.05 | 0.52±0.00 | N.D. | NS |
| ***P_sample_*** |  | * | NS | NS |  |
| L-Limonene | **PB** | 1.31±0.23 | 35.05±9.27 | 22.61±4.63 | *** |
|  | **SC** | 1.93±0.42 | 28.58±3.27 | 15.28±3.43 | ** |
|  | **VP** | 10.42±4.36 | 26.53±6.71 | 6.41±0.00 | ** |
| ***P_sample_*** |  | ** | NS | * |  |

ND: Not detected. NS: Not significant. *P_day_* and *P_sample_* imply the effect of ripening time and sample type on the levels of terpenes, respectively; PB: Plastic barrel; VP: Vacuum package; SC: Sausage casing; *: *P*<0.05; **: *P*<0.01; ***: *P*<0.001

Supplementary Table S8. Miscellaneous compounds in EU PDO Erzincan Tulum cheeses ripened in different package materials

| **Volatile compounds** | **Cheeses** | **Day 1** | **Day 30** | **Day 60** | **P*_day_*** |
| --- | --- | --- | --- | --- | --- |
| ***Miscellaneous compounds*** |  |  |  |  |  |
| Methanethiol | **PB** | 1.08±0.03 | 0.46±0.51 | 0.64±0.00 | * |
|  | **SC** | 0.63±0.64 | N.D. | 1.06±0.01 | NS |
|  | **VP** | 0.99±0.00 | N.D. | 0.91±0.12 | NS |
| ***P_sample_*** |  | NS | NS | * |  |
| Dimethyl disulfide | **PB** | N.D. | 0.11±0.03 | 0.41±0.05 | NS |
|  | **SC** | N.D. | 0.57±0.00 | N.D. | NS |
|  | **VP** | N.D. | 0.35±0.00 | 0.32±0.00 | NS |
| ***P_sample_*** |  | NS | ** | * |  |
| Methyl-benzene | **PB** | 2.86±0.95 | 3.09±0.11 | 2.95±0.85 | NS |
|  | **SC** | 3.12±0.02 | 5.39±0.38 | 3.08±0.77 | NS |
|  | **VP** | 2.17±0.05 | 4.35±0.70 | 2.28±0.46 | NS |
| ***P_sample_*** |  | NS | NS | NS |  |
| Benzeneethanol | **PB** | N.D. | N.D. | 1.30±0.00 | NS |
|  | **SC** | N.D. | 0.97±0.14 | 0.66±0.00 | NS |
|  | **VP** | 0.34±0.08 | 2.27±0.00 | 1.67±0.70 | NS |
| ***P_sample_*** |  | * | ** | NS |  |

ND: Not detected. NS: Not significant. *P_day_* and *P_sample_* imply the effect of ripening time and sample type on the levels of miscellaneous compounds, respectively PB: Plastic barrel; VP: Vacuum package; SC: Sausage casing; *: *P*<0.05; **: *P*<0.01; ***: *P*<0.001
